# Metformin and adipose-derived stem cell combination therapy alleviates radiation-induced skin fibrosis in mice

**DOI:** 10.1186/s13287-023-03627-7

**Published:** 2024-01-08

**Authors:** Hamid Malekzadeh, Yusuf Surucu, Somaiah Chinnapaka, Katherine S. Yang, José A. Arellano, Yasamin Samadi, Michael W. Epperly, Joel S. Greenberger, J. Peter Rubin, Asim Ejaz

**Affiliations:** 1 Department of Plastic Surgery, University of Pittsburgh Medical Center, 3550 Terrace Street, 6B Scaife Hall, Pittsburgh, PA 15261 USA; 2 Department of Radiation Oncology, University of Pittsburgh Cancer Institute, Pittsburgh, PA USA; 3 McGowan Institute, University of Pittsburgh, Pittsburgh, USA; 4 Department of Bioengineering, Swanson School of Engineering, University of Pittsburgh, Pittsburgh, USA

**Keywords:** Radiation, Fibrosis, Metformin, Prophylactic therapy, Adipose stem cells

## Abstract

**Background:**

Radiation therapy often leads to late radiation-induced skin fibrosis (RISF), causing movement impairment and discomfort. We conducted a comprehensive study to assess the effectiveness of metformin and adipose-derived stem cells (ASCs), whether autologous or allogeneic, individually or in combination therapy, in mitigating RISF.

**Methods:**

Using a female C57BL/6J mouse model subjected to hind limb irradiation as a representative RISF model, we evaluated metformin, ASCs, or their combination in two contexts: prophylactic (started on day 1 post-irradiation) and therapeutic (initiated on day 14 post-irradiation, coinciding with fibrosis symptoms). We measured limb movement, examined skin histology, and analyzed gene expression to assess treatment efficacy.

**Results:**

Prophylactic metformin and ASCs, whether autologous or allogeneic, effectively prevented late fibrosis, with metformin showing promising results. However, combination therapy did not provide additional benefits when used prophylactically. Autologous ASCs, alone or with metformin, proved most effective against late-stage RISF. Prophylactic intervention outperformed late therapy for mitigating radiation skin damage. Co-culture studies revealed that ASCs and metformin downregulated inflammation and fibrotic gene expression in both mouse and human fibroblasts.

**Conclusions:**

Our study suggests metformin's potential as a prophylactic measure to prevent RISF, and the combination of ASCs and metformin holds promise for late-stage RISF treatment. These findings have clinical implications for improving the quality of life for those affected by radiation-induced skin fibrosis.

**Supplementary Information:**

The online version contains supplementary material available at 10.1186/s13287-023-03627-7.

## Introduction

Radiation therapy stands as a pivotal pillar in modern cancer treatment, offering potential benefits to nearly half of all cancer patients in managing their conditions [1]. While advancements in therapy applications have minimized collateral damage to healthy cells, radiation-induced damage to normal tissues remains a significant concern [2]. The skin, acting as the body's primary defense against external threats, becomes vulnerable to ionizing irradiation-induced tissue damage as a recognized late side effect of radiation therapy. Additionally, exposure to radiation stemming from accidents, conflicts, or acts of terrorism can also impact the skin. These late effects can manifest progressively and severely, leading to radiation-induced skin fibrosis (RISF). RISF is characterized by various functional and anatomical impairments, including reduced tissue elasticity, limited mobility, atrophy, telangiectasias, xerosis, impaired wound healing, alopecia, follicle and sebaceous gland fibrosis, pigment alterations, and necrosis [3, 4].

RISF results from a complex cascade of events involving the dysregulation of several cellular and non-cellular factors following radiation injury. This dysregulation leads to excessive production and deposition of extracellular matrix (ECM) at the site of radiation injury [5]. Initial radiation exposure damages the epithelial and endothelial cells lining the vasculature, resulting in hypocellularity and hypoxia, characterized by elevated levels of GM-CSF and M-CSF [6]. Myofibroblasts, key players in the fibrogenesis process, arise from resident or migratory fibroblasts and are associated with tissue repair and fibrosis [7, 8]. In normal wound healing, myofibroblasts undergo apoptosis. However, radiation injury perpetuates an inflammatory signal cycle that drives fibroblast differentiation into myofibroblasts, which are responsible for excessive ECM deposition [9]. The profibrotic role of TGF-β1 is evident through observations that exogenous injection or genetic overexpression of TGF-β1 in mice leads to fibrosis development [10]. Triggered events like wound healing or ionizing radiation exposure induce rapid TGF-β1 activation, which plays a pivotal role in regulating ECM remodeling by balancing synthesis and degradation [11, 12]. Irradiation disrupts this balance by concurrently upregulating genes responsible for ECM synthesis while down-regulating matrix-degrading proteases [13].

There are currently no effective treatments for managing RISF. Use of vitamin E has shown clinical benefits with a mean regression of 40% after 6 months of treatment [14]. Other study demonstrated that the clinical benefits could only be achieved at high doses of vitamin E, which has reflected side effects in terms of increase in all-cause total mortality [15]. A combined use of vitamin E and pentoxifylline, a methylxanthine derivative, has shown promising results [16, 17]. A 2022 meta-analysis was unable to determine the effectiveness of pentoxifylline and vitamin E [18]. Autologous fat grafting has demonstrated promise in preclinical and clinical settings for post-radiation damage [19]. In 2007 using autologous fat, Rigotti et al. reported first clinical evidence of improved fibrosis and Late Effects Normal Tissue Task Force (LENT)-Subjective, Objective, Management, Analytic (SOMA) scores, attributing these improvements to enhanced vascularization and successful healing of refractory irradiated chest wounds [20]. In irradiated NOD/SCID mice, fat grafting reduced skin injury and expedited wound healing, accompanied by evidence of adipose-derived stem cells (ASCs) migration to the radiation injury site [21, 22]. It is hypothesized that ASCs mitigate fibrosis by antagonizing mediators of the TGF-β pathway through paracrine factors. Recent publication from our laboratory in 2019 and 2023 suggests that hepatocyte growth factor secreted by ASCs plays a role in mediating RISF in mice [23, 24].

Metformin, a member of the biguanide family of antidiabetic drugs, is a primary treatment for type 2 diabetes. It has demonstrated its potential to inhibit the TGF-β signaling pathway during myofibroblast differentiation and cardiac fibrosis [25]. Additionally, metformin has shown promise in reducing collagen accumulation in bleomycin-induced pulmonary lung fibrosis and mitigating pulmonary fibrosis induced by radiation in a mouse model [26, 27]. In 2018 a comprehensive study demonstrated that oral administration of metformin significantly reduced radiation-induced skin thickening and collagen accumulation [28]. Mechanistically, metformin suppresses radiation-induced skin injuries by modulating the expression of FOXO3 through PIK3r1 [28].

In this investigation, we assessed the potential of various treatment approaches for both preventing and treating RISF. These approaches included the use of autologous or allogeneic adipose-derived stem cells individually, metformin as a standalone therapy, or their combined application with the hypothesis that two different therapy approaches having different modes of action might have a synergistic effect.

## Methods

### Experimental model and study participant details

All experimental procedures involving C57BL/6 and FVB mice were conducted in strict adherence to the guidelines and regulations established by the Association for Assessment and Accreditation of Laboratory Animal Care International (AAALAC). The care and treatment of the animals followed the principles outlined in the National Institutes of Health Guide for the Care and Use of Laboratory Animals. Ethical approval for all experiments was obtained from the University of Pittsburgh Institutional Animal Care and Use Committee (IACUC). The irradiation of mice was executed following previously detailed protocols [29]. No exclusion was performed. Weight loss and severe ulceration were set as the end points before the planned sacrifice. Before irradiation, the mice's legs were carefully shaved to ensure uniform exposure. For the irradiation procedure, mice were first anesthetized through the intraperitoneal injection of 1.25 mg/kg of Nembutal, a well-established anesthetic agent provided by Lundbeck in Copenhagen, Denmark. Subsequently, the mice were meticulously positioned within the radiation field and securely immobilized using tape to achieve precise targeting for irradiation. To generate β-irradiation burns, a 6-MeV electron beam emitted from a Varian 23EX linear accelerator (Varian Medical Systems, Inc., Palo Alto, CA) was employed.

During the irradiation process, a 1-cm-thick bolus was thoughtfully positioned to prevent the deep penetration of radiation, thereby ensuring that only the shaved upper right rear leg of each mouse was exposed to the irradiation field, which measured 25 × 25 cm. To achieve a consistent and predetermined dosage, all monitor units were meticulously calculated by incorporating the appropriate applicator factor and cutout factors, thereby ensuring that the dose delivered to the mouse skin was precisely 40 Gy. This careful setup and execution of the irradiation protocol served as a crucial foundation for our subsequent investigations into radiation-induced skin injuries and the evaluation of potential mitigating interventions. Animals were randomized for treatment and control groups. Confounders were not controlled. Animals were euthanized at the end of the study design point using CO_2_ infusion in a CO_2_ line-connected box at a gas infusion rate of 1.5–3.5 L/min. Once the animals cease respiration for 5–10 min, the euthanasia is confirmed by cervical dislocation.

### Leg contracture measurements

To assess the extent of leg movement, we employed a protractor as a measuring tool, systematically quantifying the degree of motion, as previously described [29]. Before conducting the measurements, the mice were anesthetized using isoflurane to ensure they remained immobile during the assessment. A protractor was securely affixed to the bench adjacent to the nose cone responsible for delivering isoflurane anesthesia. This ensured that the protractor remained stable and in the optimal position for precise measurements. To begin the measurement, the right knee of the anesthetized mouse was carefully positioned at the center of the protractor. The knee was firmly held in place using the experimenter's left hand to maintain stability. With the knee secured, the experimenter used their right hand to dorsiflex the foot of the mouse. This motion was achieved by gently manipulating the foot using the index and thumb (pollex) fingers. As the foot was dorsiflexed, the degree of extension was noted and recorded. This value was determined by reading the measurement indicated by the toes on the protractor. The leg contracture analysis was performed as a non-blinded approach.

### Histological evaluation of skin damage and collagen deposition

Tissue samples were meticulously preserved by immersion in a 10% formalin solution, subsequently embedded in paraffin, and then sectioned into thin 5-μm slices. To evaluate the relative levels of collagen within these tissue samples, we employed Masson's trichrome staining, a well-established technique for highlighting collagen-rich areas within tissues. Subsequently, the stained slides were subjected to a thorough examination by a skilled pathologist who conducted a comprehensive assessment, particularly focusing on the identification of any signs indicative of fibrosis. This meticulous examination allowed us to gain insights into the presence and extent of fibrotic changes within the tissue samples. To further evaluate the condition of the skin, separate sections were subjected to hematoxylin and eosin staining [30, 31]. This staining method enabled us to visualize the skin's microscopic structure, facilitating an assessment of any visible signs of skin damage or alterations. In particular, the Masson's Trichrome stained skin sections underwent a detailed scoring process, conducted in a blinded manner as per established guidelines [6]. This scoring encompassed the evaluation of various parameters, including inflammation, fibrosis, vascularity, and cellular alterations. By adhering to these well-defined assessment criteria, we were able to objectively quantify and characterize the extent of these histological changes within the skin samples. These histopathological analyses served as a crucial component of our research, allowing us to draw meaningful conclusions regarding the effects of different interventions on radiation-induced skin injuries.

### Isolation of adipose-derived stem cells

Subcutaneous adipose tissue was meticulously collected from the mice, ensuring sterile conditions throughout the procedure, to maintain the integrity of the samples. The collected tissue was then carefully cut into smaller pieces, each weighing approximately 1–2 mg. These tissue fragments were subjected to a digestion process using a specialized digestion buffer, which consisted of Hank's Balanced Salt Solution (HBSS) sourced from Fisher Scientific in Massachusetts, USA. The buffer was supplemented with 200 units per milliliter of collagenase (CLS Type I, procured from Worthington Biochemical Corp. in Lakewood, NJ) and 2% w/v bovine serum albumin (BSA) from Sigma, USA. The digestion process took place under constant stirring for 60 min, maintaining a temperature of 37 °C and a rotational speed of 450 revolutions per minute. The ratio used was 1 mg of adipose tissue to 3 ml of digestion buffer. Following the digestion process, the dispersed tissue underwent a centrifugation step lasting 5 min at 200 relative centrifugal force (RCF) and was conducted at room temperature. This centrifugation facilitated the separation of floating adipocytes from the sedimented stromal vascular fraction (SVF). The floating adipocytes were carefully aspirated, leaving behind the pelleted SVF. The SVF was then resuspended in an erythrocyte lysis buffer sourced from Fisher Scientific, MA, USA, and allowed to incubate for 2 min at room temperature. This step was essential for removing any remaining tissue debris. To further refine the cell suspension and eliminate residual cell aggregates, the SVF was passed through a nylon mesh filter with a pore size of 100 μm, obtained from BD, USA. Subsequently, another round of centrifugation lasting 5 min at 200 RCF was performed. The resulting pelleted SVF was then resuspended in Dulbecco's Modified Eagle Medium (DMEM) from Fisher Scientific, MA, USA, supplemented with 10% fetal bovine serum (FBS) sourced from Sigma. To ensure the complete removal of any remaining cell aggregates, the SVF suspension was once again filtered, this time through a 70 μm mesh. The adherent cell population that remained after this meticulous process was referred to as the adipose-derived stromal cell (ASC) fraction. This highly purified ASC fraction was used for all subsequent studies. It is important to note that cells in passages 4–5 were employed in the experiments conducted as part of this study, ensuring consistency and reliability in the results obtained. 3 × 10^6^ autologous or allogeneic ASCs were used per injection.

### Transwell co-culture

A Transwell system (0.4 µm pore size, Polyester (PET) membrane; Corning, USA) was employed. Mouse or human adipose-derived stem cells were cultured in the top transwell basket compartment, while mouse (L929 cells, ATCC, USA) or human foreskin fibroblasts (ATCC, USA) were cultured in the lower compartment. Fibroblasts were grown to confluence and irradiated using a 10Gy radiation dose. Twenty-four hours post-irradiation 3 × 10^5^ ASCs or 20 µM metformin or both were added to the upper transwell basket. Co-culture was maintained for 72 h. Cells were lysed, and total RNA was isolated using TRIzol (Sigma, USA) reagent following the manufacturer’s protocol. cDNA was synthesized and the expression of profibrotic gene battery (IL-6, CTGF, Collagens 1 and 3) was analyzed using gene-specific primers employing quantitative real-time PCR.

### Real-time PCR

RNA was extracted from mouse skin or cultured cells using the TRIzol reagent following the manufacturer’s instructions. RNA was reverse transcribed using a High Capacity cDNA Reverse Transcription Kit (Applied Biosystems, USA) according to the manufacturer’s protocol. Using cDNA as a template and gene-specific primers (Millipore Sigma, USA) expression of IL-1, IL-6, TGF β1, TNFα, NF-κB, and Collagens 1–6 was quantified by quantitative real-time PCR using the Eppendorf Realplex2 Mastercycler. Data for each gene transcript were normalized by calculating the difference (∆Ct) from the Ct-housekeeping and Ct-Target genes. The relative increase or decrease in expression was calculated by comparing the reference gene with the target gene calculated by comparing the reference gene with the target gene (∆∆Ct) and using the formula for relative expression (= ^2∆∆Ct^).

### Quantification and statistical analyses

All data are reported as the mean ± standard deviation to assess the between-subject variability of the measured outcome. The student’s T-test and Analysis of variance (ANOVA) test was performed where applicable using GraphPad Prism software. Animal numbers were based on our previous studies [24].

### Use of generative AI and AI-assisted technologies in the writing process

During the preparation of this manuscript, the author(s) used [ChatGPT3.5] to assist with English language use. After using this tool/service, the author(s) reviewed and edited the content as needed and take(s) full responsibility for the content of the publication.

## Results

### Prophylactic use of ASCs or metformin reduces late skin and muscle fibrosis in irradiated hindlimbs

Our mouse model for studying RISF entails exposing C57BL/6 mice to focused irradiation at 40 Gy directed at the hindlimb soft tissue. This irradiation regimen consistently results in histologically evident fibrotic tissue within 14–21 days, accompanied by a measurable reduction in range of motion. Additionally, this radiation-induced fibrotic response is associated with the upregulation of key fibrosis-related genes, including TGF-β, CTGF, TNF, IL1, NF-kB, and Col1-6. [23, 29]

**Fig. 1:**
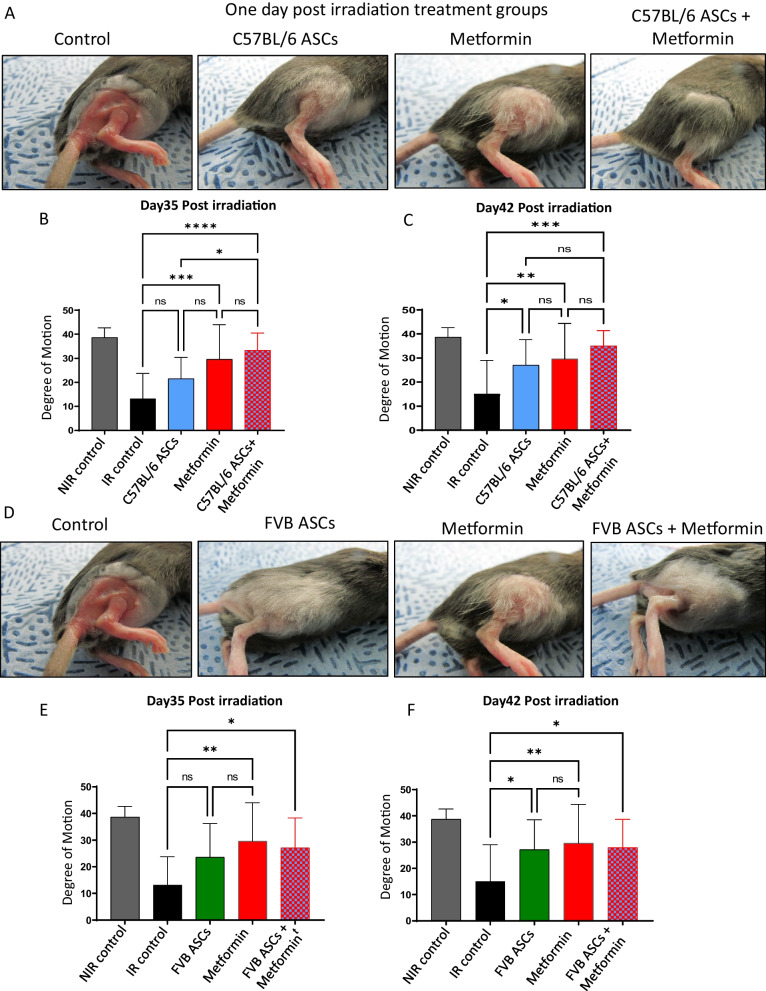
Impact of ASCs, metformin, or combination prophylactic therapy on functional outcomes linked to RISF. **A** Skin architecture at day 42 post 40Gy irradiation. Local injection of 3 × 10^6^ autologous ASCs (C57BL/6 origin ASCs injected in irradiated C57BL/6 host) on day 1 post-irradiation or 100 mg/kg body weight metformin was injected 3 times a week starting day 1, or a combination of both ASCs and metformin or saline (control) was injected. **B** and **C** The degree of leg motion was measured on days 35 (**B**), and 42 (**C**) post-irradiation and plotted (*n* = 8–10 per group). **D** Skin architecture at day 42 post 40Gy irradiation. Local injection of 3 million allogeneic ASCs (FVB origin ASCs injected in irradiated C57BL/6 host) on day 1 post-irradiation or 100mg/kg body weight metformin was injected 3 times a week starting day 1, or a combination of both ASCs and metformin or saline (control) was injected. **E** and **F** The degree of leg motion was measured on days 35 (**E**), and 42 (**F**) post-irradiation and plotted (*n* = 8–10 per group). *P* value < 0.05 = ^*^, < 0.005 = ^**^, < 0.0005 = ^***^, < 0.00005 = ^****^

To assess potential therapeutic strategies for RISF, we investigated the prophylactic effects of autologous ASCs by administering 3 × 10^6^ cells, isolated from C57BL/6 mice, subcutaneously through rigotomy on day 1 post-irradiation. Alternatively, intraperitoneal treatment of metformin at a dose of 100 mg/kg, administered three times per week beginning on day 1, was explored. In addition, we examined the combined application of autologous ASCs and metformin in our model. Our results indicated notable reductions in soft tissue fibrosis, hair loss, and enhanced passive limb excursion movements in mice treated prophylactically compared to control mice receiving saline injections. These improvements were particularly evident at days 35 and 42 post-irradiation (Fig. 1A–C and Additional file 1: Fig. S1A–D). Although the extent of mitigation between the ASCs and metformin groups did not exhibit significant differences, the metformin group displayed relatively better limb movements and skin texture (Fig. 1B, C and Additional file 1: Fig. S1B and C). Notably, no synergistic effects were observed between ASCs and metformin when administered prophylactically (Fig. 1B and C).

Nonetheless, the procurement of autologous ASCs entails a multifaceted process, necessitating patient anesthesia and potential post-procedure trauma. It is essential to acknowledge that numerous cancer patients undergoing radiotherapy often possess limited fat reserves and may struggle with persistent psychological distress from their cancer treatment. These factors render them less ideal candidates for the collection of adipose tissue. Moreover, the practical feasibility of employing autologous ASCs as a countermeasure scenarios involving mass radiation exposure remains constrained. Consequently, we explored an alternative avenue, considering the utilization of allogeneic ASCs as a strategy for mitigating RISF.

In our investigation, we conducted a comparative assessment of the effectiveness of allogeneic ASCs derived from FVB mice in ameliorating irradiation-induced damage in C57BL/6 mice. This assessment was juxtaposed with treatments employing saline as a control, metformin, or a combination of FVB ASCs and metformin. Interestingly, our findings unveiled that allogeneic ASCs treatment exhibited a similar pattern of mitigation dynamics as autologous ASCs, resulting in significant preservation of skin texture, hair, and limb mobility (Fig. 1D–F and Additional file 1: Fig. S1E). When juxtaposed against treatment with metformin alone or the combination therapy of ASCs and metformin, we observed no significant deviations (Fig. 1D–F and Fig. S1C, E, and F). In summary, our above findings highlight the potential of both metformin and ASCs prophylactic administration, whether autologous or allogeneic, to effectively mitigate radiation-induced skin damage.

### Prophylactic use of ASCs or metformin prevents fibrosis and inflammation and maintains vascular integrity and cellular architecture in the irradiated skin

**Fig. 2:**
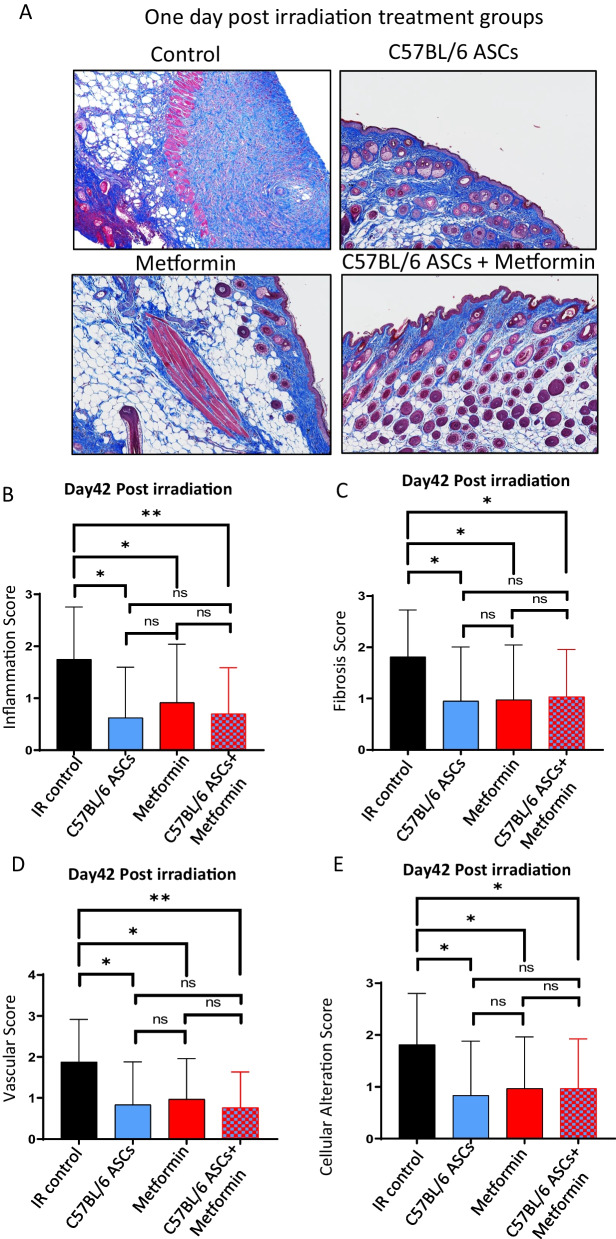
Impact of autologous ASCs, metformin, or combination therapy on histological outcomes linked to RISF. **A** Skin was surgically excised from a 40Gy irradiated field and histologically processed sections were stained with Masson’s Trichrome stain. **B–E** Histologically stained sections were blindly scored for inflammation (**B**), fibrosis (**C**), vascularization (**D**), and cellular alteration (**E**). Scores were plotted (*n* = 8–10). *P* value < 0.05 = ^*^, < 0.005 = ^**^

Histological sections of irradiated skin were subjected to Masson's Trichrome staining, and a blinded scoring system was employed to assess various parameters, including inflammation, fibrosis, vascularity, and cellular alterations, using established guidelines [6]. The scores obtained shed light on the efficacy of autologous ASCs, metformin, or combination therapy in decreasing fibrosis and inflammation in irradiated skin (Fig. 2A–C and Additional file 1: Fig. S2A–D). In addition, our histological evaluation revealed preservation of vasculature and cellular architecture in treatment groups compared to control (Fig. 2D and E). Consistent with our observations, which did not indicate a significant improvement in limb mobility when comparing single therapy to the combined regimen, we did not observe any substantial changes in the histological outcomes with combination therapy as compared to the application of ASCs alone or metformin alone.

**Fig. 3:**
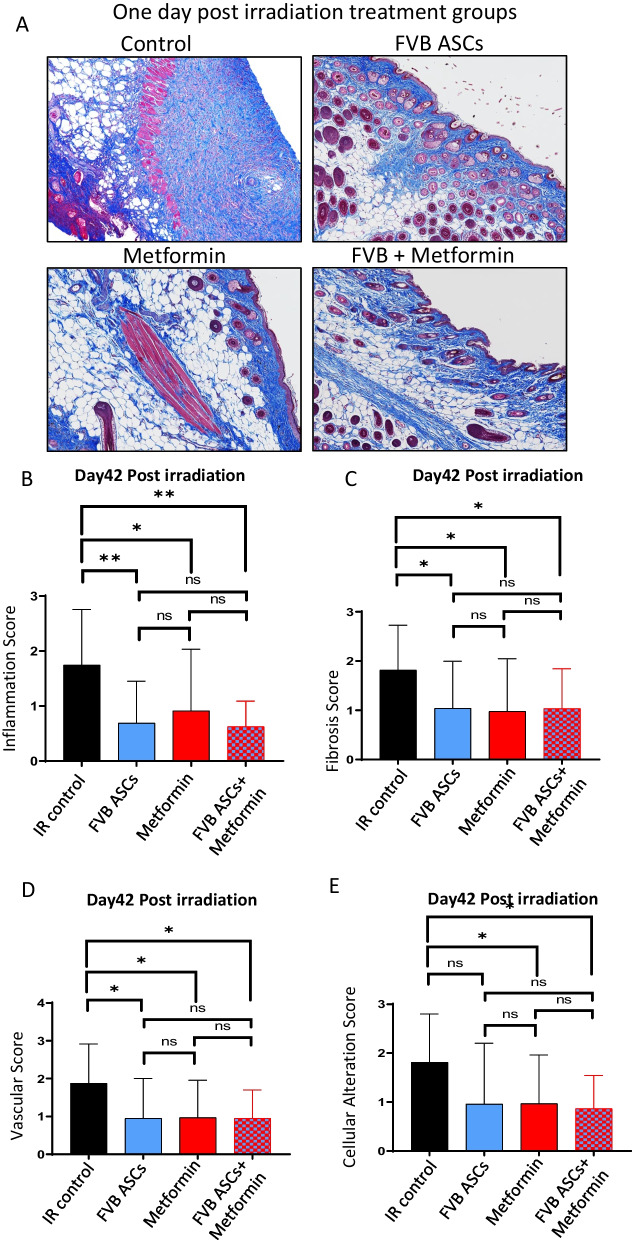
Impact of allogeneic ASCs, metformin, or combination therapy on histological outcomes linked to RISF. **A** Skin was surgically excised from a 40Gy irradiated field and histologically processed sections were stained with Masson’s Trichrome stain. **B–E** Histologically stained sections were blindly scored for inflammation (**B**), fibrosis (**C**), vascularization (**D**), and cellular alteration (**E**). Scores were plotted (*n* = 8–10). *P* value < 0.05 = ^*^, < 0.005 = ^**^

Furthermore, we extended our evaluation to compare histological outcomes in terms of fibrosis, inflammation, vascularity, and cellular alterations following the administration of allogeneic ASCs, metformin alone, or a combination of allogeneic ASCs and metformin therapy. Our findings demonstrated that prophylactic use of allogeneic ASCs effectively prevented skin epithelium thickening, inflammation, devascularization, and cellular disintegration in irradiated skin (Fig. 3A–E and Additional file 1: Fig. S2C, 2E, and 2F). However, in comparison to autologous ASCs therapy, the combined therapy of allogeneic ASCs and metformin failed to exhibit additive benefits in preserving radiation-induced skin damage at the histological level. In conclusion, these results support the observation that prophylactic use of either autologous or allogeneic ASCs, as well as metformin monotherapy, effectively reduces fibrosis in irradiated skin. Of note, the same control animals were used for the autologous and allogeneic application as part of a larger experiment.

### Prophylactic use of ASCs or metformin reduces inflammation- and fibrosis-associated gene expression in irradiated skin

**Fig. 4:**
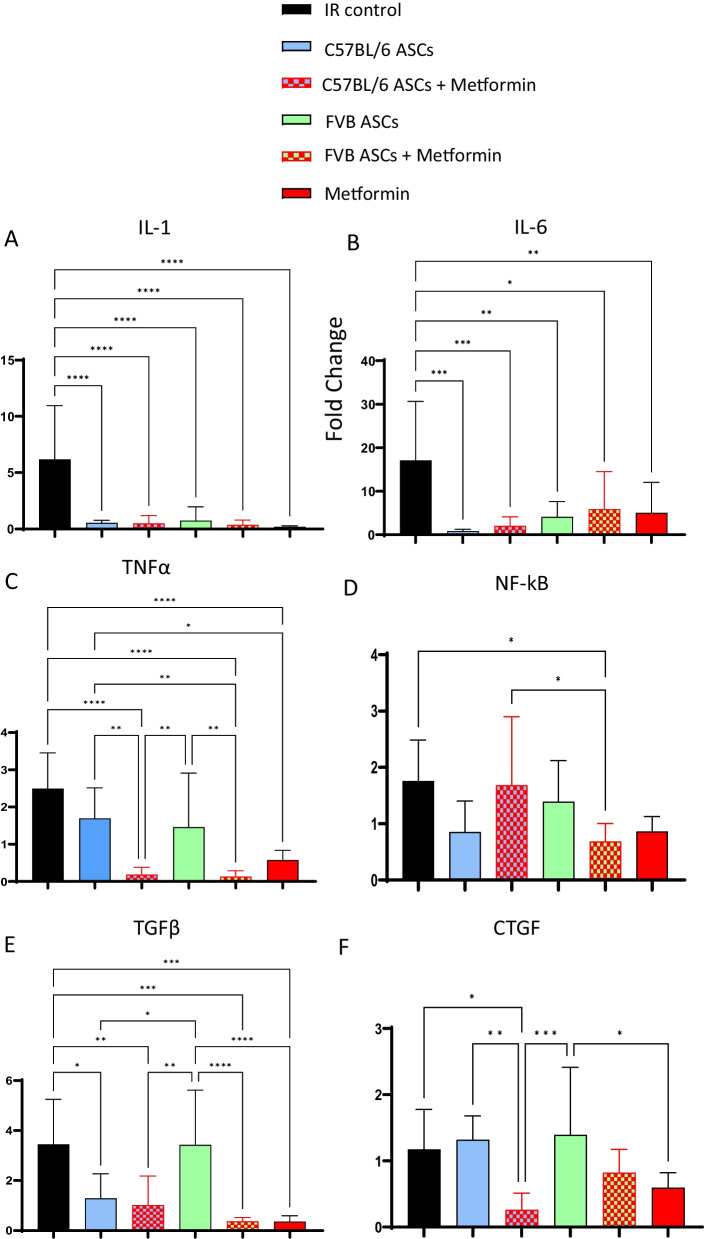
Effect of different therapeutic interventions on gene expression linked to RISF. Expression of genes related to inflammation (**A**–**D**) (IL-1, IL-6, TNFα, and NF-ĸB) and fibrosis (**E**–**F**) (CTGF and TGF-β) was analyzed in the skin of 40Gy irradiated and differentially treated mice: irradiated control, C57BL/6 ASCs, C57BL/6 ASCs + metformin, FVB ASCs, FVB ASCs + metformin, and metformin on day 42 post-irradiation using real-time quantitative PCR (*n* = 8–10). *P* value < 0.05 = ^*^, < 0.005 = ^**^, < 0.0005 = ^***^, < 0.00005 = ^****^

In the context of radiation-induced fibrosis, inflammation and fibrosis represent the hallmark features of this condition [6]. Next, we investigated the molecular aspects of this phenomenon by analyzing quantitative real-time PCR data derived from skin tissues. These analyses unveiled compelling insights into the impact of various prophylactic treatments on the expression of key inflammatory genes and mediators associated with fibrosis. Firstly, our findings indicated a significant reduction in the expression of inflammatory genes IL-1 and IL-6 upon prophylactic administration of autologous ASCs, allogeneic ASCs, metformin, or the combined therapy of ASCs and metformin (Fig. 4A and B). Moreover, metformin, either administered alone or in conjunction with ASCs, demonstrated a substantial decrease in the expression of TNFα and NF-ĸβ in irradiated skin (Fig. 4C and D). TGF-β, recognized as the central mediator in the development and progression of fibrosis [31], exhibited significantly lower expression levels in irradiated skin when treated with autologous ASCs, metformin as a standalone treatment, or in combination, particularly at day 42 post-irradiation (Fig. 4E). Intriguingly, the prophylactic use of allogeneic ASCs alone did not lead to any discernible changes in TGF-β expression in irradiated skin (Fig. 4E). Another pivotal player in the fibrosis landscape, the CTGF gene, was effectively regulated by metformin, as evidenced by our data (Fig. 4F). Our comprehensive molecular analysis underscores the multifaceted effects of these prophylactic interventions on key inflammatory and fibrotic mediators in irradiated skin, providing valuable insights into their mechanisms of action and potential therapeutic implications.

### ASCs and metformin reduce inflammation and fibrosis gene expression in irradiated fibroblasts

**Fig. 5:**
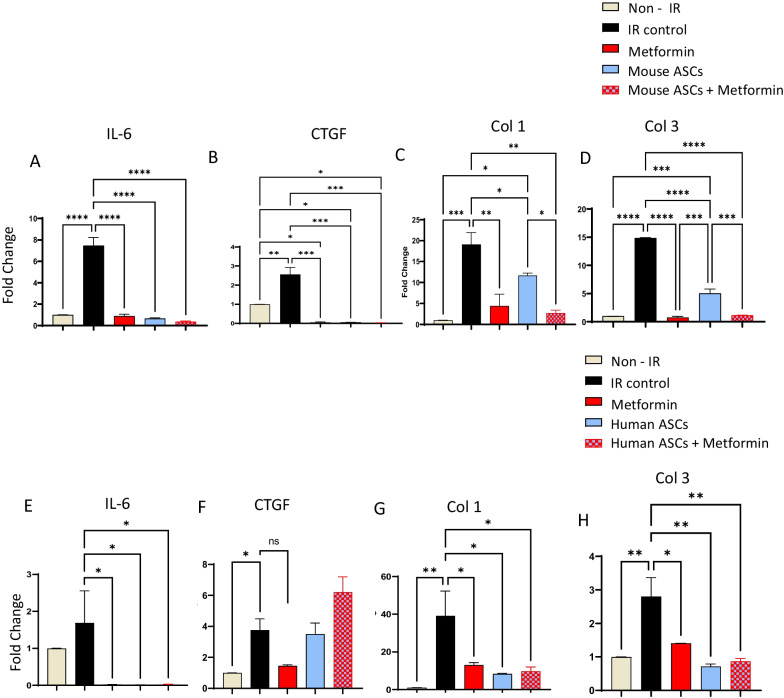
Effect of ASCs and metformin on irradiated fibroblasts in transwell co-culture. **A**–**D** Mouse fibroblast cell line L929 cells were grown to confluence in the lower chamber of the transwell plate, irradiated with 10Gy irradiation dose, and mouse ASCs, metformin, or both were added 24 h. post-irradiation for 72 h. RNA was isolated from the fibroblasts and analyzed by quantitative RT-PCR for the expression of IL-6 (**A**), CTGF (**B**), Col1 (**C**), and Col3 (**D**). **E–H** Human foreskin fibroblasts were grown to confluence in the lower chamber of the transwell plate, irradiated with 10Gy irradiation dose, and human ASCs, metformin, or both were added 24 h post-irradiation for 72 h. RNA was isolated from the fibroblasts and analyzed by quantitative RT-PCR for the expression of IL-6 (**E**), CTGF (**F**), Col1 (**G**), and Col3 (**H**). *P* value < 0.05 = ^*^, < 0.005 = ^**^, < 0.0005 = ^***^, < 0.00005 = ^****^

Fibroblasts are pivotal players in the process of fibrosis, as they perpetuate inflammatory signals and unleash excessive extracellular matrix deposition in response to radiation-induced damage. To gain deeper insights into the precise mechanisms underlying the mitigatory effects of ASCs, metformin, or their combined application, we conducted transwell co-culture studies. These investigations were designed to assess the direct impact of ASCs and metformin, either individually or in combination, on the modulation of inflammation and the expression of genes associated with fibrosis in irradiated fibroblasts. In our transwell co-culture studies employing mouse-origin fibroblasts and ASCs, we made a noteworthy observation. Both ASCs and metformin, when administered alone or in tandem, demonstrated a remarkable ability to downregulate the expression of key inflammatory genes, including IL-6, and fibrotic genes such as CTGF, Col1, and Col3 (Fig. 5A–D). This finding highlights the capacity of these therapeutic agents to directly influence the fibrogenic signature of irradiated fibroblasts.

Expanding the scope of our investigation, we extended these transwell co-culture studies to encompass human-origin fibroblasts subjected to irradiation. In this setup, we utilized irradiated human foreskin fibroblasts as the target cells, while human ASCs and metformin served as the mitigating agents. Consistent with our murine study, we observed a significant reduction in the expression of inflammation-related gene IL-6, as well as fibrosis-associated genes CTGF, Col1, and Col3 (Fig. 5E–H). Remarkably, the synergistic effects stemming from the combination of ASCs and metformin did not yield a significant advantage over the individual use of these agents. Collectively, the outcomes of these direct interaction studies underscore the profound impact of both therapeutic agents on irradiated fibroblasts, leading to a reduction in their fibrogenic signature. This evidence reinforces the potential of ASCs and metformin as therapeutic strategies capable of directly modulating the fibrotic response in irradiated tissues.

### A combination of ASCs and metformin offers significantly enhanced amelioration of advanced-stage radiation damage

In our exploration of the dynamics underlying radiation-induced skin injuries, we observed a distinct pattern characterized by a peak in inflammation and the expression of fibrotic genes, typically occurring around day 14 following irradiation exposure. By this time point, visible signs of skin injury had manifested, and the mice began to exhibit a decline in limb motion activity [23, 29]. Drawing from our above finding, which demonstrated the efficacy of ASCs in ameliorating late-stage fibrosis in mice, we next sought to investigate the potential of metformin in mitigating late radiation-induced damage, a facet that had remained relatively unexplored. Hence, we performed a comparative study, evaluating the therapeutic benefits of ASCs, metformin, and their combination in addressing late-stage damage induced by irradiation.

**Fig. 6:**
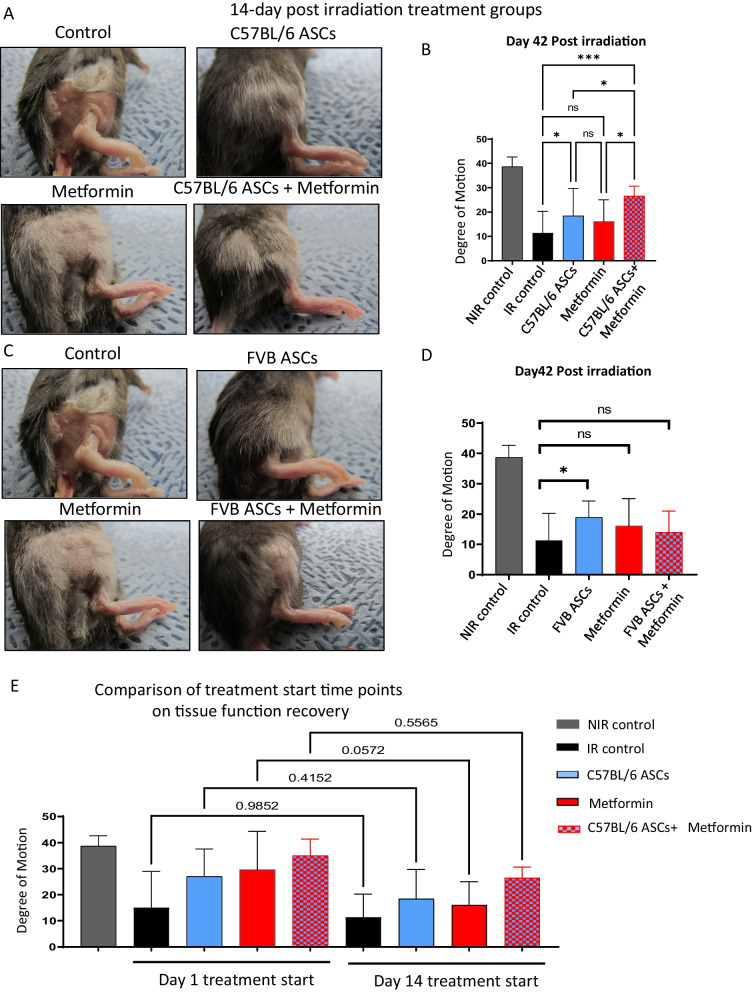
Impact of ASCs, metformin, or combination late-stage therapy on functional outcomes linked to RISF. **A** Skin architecture at day 42 post 40Gy irradiation. Local injection of 3 million autologous ASCs (C57BL/6 origin ASCs injected in irradiated C57BL/6 host) on day 14 post-irradiation or 100mg/kg body weight metformin was injected 3 times a week starting day 14, or a combination of both ASCs and metformin or saline (control) was injected. **B** The degree of leg motion was measured on day 42 (**B**) post-irradiation and plotted (*n* = 8–10 per group). **C** Skin architecture at day 42 post 40Gy irradiation. Local injection of 3 million allogeneic ASCs (FVB origin ASCs injected in irradiated C57BL/6 host) on day 14 post-irradiation or 100mg/kg body weight metformin was injected 3 times a week starting day 14, or a combination of both ASCs and metformin or saline (control) was injected. **D** The degree of leg motion was measured on day 42 (**D**) post-irradiation and plotted (*n* = 8–10 per group). **E** comparison of prophylactic and late-stage therapy on the outcomes of mitigation. *P* value < 0.05 = ^*^, < 0.005 = ^**^, < 0.0005 = ^***^

In this experimental setup, we administered a single dose of ASCs at day 14 post-irradiation or initiated metformin treatment with three intraperitoneal injections per week, also commencing at day 14. The combination therapy group received both ASCs injection on day 14 and metformin treatment, following the same schedule. Our weekly imaging assessments of the irradiated skin revealed that the combination therapy of ASCs and metformin yielded the most favorable outcomes in terms of wound healing, skin softening, and hair retention (Additional file 1: Fig. S3A–D), surpassing the effects observed with ASCs alone and metformin alone. Importantly, the functional evaluation of limb excursion revealed a significantly enhanced recovery in limb motion in the combination therapy group compared to both the control and single therapy regimens (Fig. 6A and B). Moreover, ASCs exclusive therapy also demonstrated a significantly superior functional outcome compared to the control group of irradiated mice. To further expand the scope of our study, we extended our investigations to assess the potential of allogeneic ASCs in mitigating late-stage radiation damage. The analysis of limb excursion revealed that allogeneic ASCs administered as a standalone therapy led to an improvement in limb motion, albeit the combination therapy failed to exert any notable effects (Fig. 6C and D). Additionally, the visible improvements in skin texture resulting from allogenic cell therapy were less pronounced in comparison to the outcomes achieved with autologous ASCs and the combination of autologous ASCs and metformin, which exhibited an exceptionally pronounced effect on the skin.

Crucially, as part of our comprehensive analysis, we compared the outcomes of prophylactic and late-stage treatments using different mitigators for radiation-induced fibrosis. Our results unequivocally demonstrated that the outcomes from prophylactic therapy interventions were notably superior compared to late-stage treatments (Fig. 6E). In light of these findings, we posit that a combination of ASCs and metformin represents a promising therapeutic approach for mitigating the late side effects of irradiation. This approach has the potential to significantly enhance the quality of life for individuals who have undergone radiation therapy. Furthermore, a prophylactic approach is superior in achieving an improved outcome from irradiation damage.

## Discussion

Autologous fat grafting has emerged as a valuable tool in effectively mitigating RISF. Notably, autologous fat grafts enriched with ASCs have found routine application in reconstructive surgical procedures [32, 33]. Clinical evidence in published studies has substantiated the utility of autologous fat grafts in addressing the consequences of radiotherapy-induced tissue damage [32]. These studies have reported tangible improvements in fibrosis and scores on the Late Effects Normal Tissue Task Force-Subjective, Objective, Management, Analytic (LENT-SOMA) scale, primarily attributed to enhanced vascularization and improved hydration of fibrotic tissues, with a significant role played by ASCs [20]. Further validation of these initial observations has been provided by subsequent investigations, particularly in the context of childhood facial cancer treatment trials [34–36]. These studies have consistently demonstrated enhancements in skin texture and softening of fibrotic tissues, outcomes attributed to the anti-inflammatory and regenerative cytokines produced by adipose-derived stem cells residing within adipose tissue [23, 24]. Our recent research has also underscored the critical role of hepatocyte growth factor (HGF) secretion by ASCs in mitigating RISF [24]. Two 2023 studies from Dr. Yoshimura laboratory and our laboratory have demonstrated successful prophylactic use of adipose tissue and adipose tissue-derived products for mitigation of RISF [24, 37]. In addition to ASCs, mesenchymal stem cells from other tissue sources like bone marrow have been therapeutically used in animal models and in clinics [38]. In addition to RISF mitigation, ASCs has been applied for treating burn, diabetic, traumatic and thermal wounds [39, 40].

Adipose tissue-based therapies can be applied in different formulations. Adipose tissue enriched with stromal vascular fraction (SVF) or plasma has clinically shown benefits in graft retention and clinical outcome of RISF [20, 41]. Adipose tissue SVF is a heterogenous cell population consisting of 50–70 percent ASCs [42]. For clinical applications, SVF can be isolated from adipose tissue for autologous application using enzymatic and non-enzymatic digestion techniques [43]. Both techniques have their pros and cons. Enzymatic digestion employing collagenase enzyme results in high yield and purified cell suspension, but it has disadvantage of being expensive, time-consuming, and inconsistent in outcomes due to enzymes’s lot-to-lot variations. Non-enzymatic methods for SVF isolation use mechanical or physical forces to disrupt the adipose tissue matrix integrity and periadventitial structures [43]. Non-enzymatic digestions are cost-effective, rapid, minimally manipulated, and xeno free. The associated disadvantages include lower yield, mechanical stress, and significant amount of tissue debris [43]. We used enzymatic digestion for isolating SVF in this study. Fat graft retention and changes can be assessed volumetrically using ultrasound and magnetic resonance imaging, while clinical outcomes can be assessed using Equipe-evaluation and patient self-evaluation [41, 44]. The treatment effect of fat grafting on fibrosis outcome is routinely evaluated clinically using the Patient and Observer Scar Assessment Scale (POSAS) or Late Effects of Normal Tissue-SOMA scale (LENT-SOMA scale) [45].

Metformin, a widely employed antidiabetic medication, exerts its effects through a multifaceted mechanism involving both AMP-activated protein kinase (AMPK)-dependent and AMPK-independent pathways, achieved by inhibiting mitochondrial respiration [46]. Activation of the AMPK pathway is associated with the development of radiation-induced fibrosis by activating the TGF-β pathway in different tissues and organ systems [28]. Metformin can selectively inhibit AMPK-mediated TGF-β pathway signaling thus mitigating the fibrotic process in vital organs such as the heart, kidneys, and lungs [47–49]. Additionally, metformin has demonstrated efficacy in ameliorating bleomycin-induced skin fibrosis [50]. Recent research has highlighted the benefits of oral metformin administration in reducing skin thickening, collagen accumulation, and the downregulation of inflammation and fibrosis-related gene expression [28]. Investigating the underlying mechanism, the results demonstrated that metformin reduces radiation-induced FOX3 expression, a factor known to drive the TGF-β pathway. Although TGF- β1 has a role in inhibition of cell growth and immunosuppression, its role in regulation of extracellular matrix depositions designate it as a key player in fibrogenesis [51]. The profibrotic role of TGF- β1 is supported by the observation that both exogenous injection of TGF- β1 or genetic manipulation to overexpress TGF- β1 in mice result in establishment of fibrosis [10].

Therefore, in our current investigation, we sought to evaluate and compare the mitigatory potential of ASCs, metformin, and a combination of both agents, under the hypothesis that their distinct mechanisms of action might synergize to yield improved outcomes in mitigating RISF. Prior studies have predominantly focused on the prophylactic administration of metformin immediately following irradiation, a stage when no overt signs or symptoms of fibrosis are apparent [28]. Drawing from our previous research findings, which indicated that anatomical and molecular evidence of fibrosis becomes apparent by day 14 post-irradiation with a 40 Gy dose [23], we selected this specific time point as a pivotal juncture for assessing the efficacy of our treatments. Therefore, we examined the effects of metformin, either as a standalone intervention or in combination with ASCs, in both prophylactic and therapeutic contexts, commencing treatment on day 1 post-irradiation and day 14 post-irradiation, respectively. Utilizing an established mouse model of RISF [29], we embarked on a comprehensive evaluation of our treatment regimens, encompassing clinical, histological, and molecular assessments. Our primary measures included the quantification of fibrosis within the irradiated tissue, which was scrutinized through a combination of histological and molecular analyses. Among the notable observations in irradiated skin tissue were the thickening of the skin epithelium, the presence of edema, an influx of inflammatory cells to irradiated sites, and the upregulation of key molecular markers such as TGF-β, IL-1, IL-6, and CTGF. The functional readout of the RISF was a loss of limb excursion. Injection of ASCs or metformin or a combination of both at day 1 post-irradiation resulted in significant improvement in the clinical, histological, and molecular signature of RISF. We observed improvement in skin appearance, hair regrowth, and limb excursion. Importantly, our findings align with our earlier work demonstrating the efficacy of ASCs in mitigating RISF in a prophylactic context [24]. Use of iron chelator deferoxamine has also proven prophylactic efficacy [52]. Additionally, our results concur with previously published data highlighting the mitigatory potential of metformin when administered starting on day 1 post-irradiation [28].

Although our initial expectation was to observe a synergistic and improved outcome with combination therapy, our prophylactic application of ASCs and metformin failed to elicit synergistic effects in terms of histological or functional measures of RISF mitigation. ASCs, renowned for their regenerative potential, have demonstrated success in treating a variety of pathologies [53]. However, the response elicited by ASCs in a cell therapy context is intricately linked to the severity of the underlying condition. Considering our understanding of the temporal dynamics of radiation-induced pathology, it is noteworthy that the cascade of inflammation initiated shortly after radiation exposure peaks around day 14 post-exposure. This temporal consideration provides a plausible explanation for our inability to observe a synergistic effect from the combination therapy. Another potential explanation may be that the single or combination therapies have already achieved the maximum attainable recovery after exposure to a high irradiation dose.

Histological assessment of skin sections treated with ASCs, metformin, or a combination of both revealed a reduction in epithelium thickening, inflammation, and collagen deposition. On a molecular level, therapy administered on day 1 post-exposure resulted in decreased expression of proinflammatory and profibrosis genes. Reducing the levels of factors contributing to inflammation and fibrosis is pivotal in halting the progression of RISF [54]. Our co-culture experiments utilizing irradiated fibroblasts as a model demonstrated that both metformin and ASCs can directly attenuate the expression of inflammation and fibrosis-related genes in irradiated fibroblasts. Previous research from our laboratory has underscored the role of hepatocyte growth factor (HGF) in mitigating RISF [23]. ASCs are known for secreting a plethora of paracrine factors like IL-10, bFGF, VEGF, and TGFβ-3 upon stimulation. These factors along with HGF have been shown to exert antifibrotic effects involved in the attenuation of myocardial, pulmonary, hepatic, and renal fibrosis [55]. Studies in rats showed initial inflammation followed by the development of fibrosis, characterized by thickening of epithelium, frequent necrosis, and deposition of collagen [6]. Previous studies have shown the involvement of HGF in increased vascularization [56], decreased inflammation [57], downregulation of the profibrotic TGFβ-Smad signaling pathway [58], and promoting migration of bone marrow resident tissue-specific progenitor cells [59]. Similarly, metformin use has shown downmodulation of inflammation and fibrotic genes in irradiated NIH3T3 and MDF cells [28]. The antifibrotic characteristics of ASCs and metformin through different mechanism of actions justify testing the synergistic use of these agents.

Radiation-induced skin fibrosis (RISF) is not solely limited to cases involving cancer treatment but can also manifest as a consequence of exposure to radiation resulting from industrial accidents, warfare, or acts of terrorism. The heightened risk of such incidents has been exacerbated by the increased utilization of radioactive materials in various industrial sectors and military installations [60]. While autologous fat grafts enriched with autologous adipose-derived stem cells (ASCs) have demonstrated clinical efficacy in managing RISF, these products present practical limitations when it comes to addressing mass radiation exposure scenarios. Consequently, there is a pressing need to develop readily available mitigators for such situations. Our study marks the pioneering exploration of allogeneic ASCs as a potential mitigatory vehicle for RISF, either as a standalone treatment or in combination with metformin. Allogeneic ASCs, derived from a donor source different from the recipient, have never been investigated for their efficacy in mitigating radiation-induced skin damage. Our findings, for the first time, shed light on the remarkable potential of allogeneic ASCs in safeguarding the skin against radiation-induced harm when administered prophylactically. It is worth noting that our prior research has already demonstrated the ability of allogeneic ASCs to shield mice from the acute toxicities associated with total body radiation exposure [61]. In addition, allogeneic mesenchymal stem cells resulted in improvement in organ dysfunction in drug resistant SLE patients and improve osteogeneis in infants with severe hypophosphatasia [62, 63]. These results collectively underscore the promising prospects of allogeneic-based therapies in managing radiation-related toxicities, offering a new avenue of research in the field of radioprotection and radiation injury management.

The current clinical approach to managing radiation-induced skin fibrosis (RISF) typically involves initiating interventions once the visible signs and symptoms of RISF become apparent. However, our study aimed to assess the therapeutic potential of metformin in addressing well-established fibrosis by commencing metformin therapy on day 14 post-irradiation exposure. Our findings revealed that when metformin therapy was initiated relatively late after irradiation exposure, its effectiveness was notably reduced. Interestingly, a remarkable synergistic effect was observed when a combination therapy of ASCs and metformin was employed after the onset of RISF. These results strongly suggest the importance of regenerative agents like ASCs in mitigating late-stage fibrosis, emphasizing their potential in this context. Comparing the outcomes between prophylactic and therapeutic applications of ASCs, metformin, or combination therapy, our study concluded that prophylactic approaches yielded superior results. It is important to note that metformin, as one of the most widely used drugs with a well-established safety profile, emerges as a compelling candidate for clinical testing as a potential RISF mitigator. To validate its broader applicability and assess its efficacy, clinical studies designed to explore the prophylactic use of metformin as a mitigator for RISF are warranted. Such research endeavors hold the promise of improving the management and outcomes of RISF in clinical settings.

## Conclusions

In conclusion, our research endeavors have illuminated the multifaceted landscape of mitigating radiation-induced skin fibrosis (RISF). The integration of adipose-derived stem cells (ASCs) and the therapeutic application of metformin have demonstrated promising outcomes in ameliorating the clinical, histological, and molecular manifestations of RISF. Despite the initial anticipation of synergistic effects with combination therapy, our study demonstrated that combination therapy is effective in mitigating late effects of fibrosis but no synergistic effects were observed upon prophylactic application. Further our results emphasizes the critical importance of timing in the administration of regenerative agents. Prophylactic approaches, especially the administration of ASCs and metformin at the onset of radiation exposure, yielded superior outcomes in preventing and mitigating RISF. Notably, our investigation into the unprecedented use of allogeneic ASCs as a potential mitigator opens new avenues for research in managing radiation-related toxicities. Furthermore, our findings underscore the potential of metformin as a compelling candidate for clinical testing, particularly in a prophylactic context, given its well-established safety profile. As we advance our understanding of RISF and explore innovative interventions, our research contributes to the evolving landscape of radioprotection and radiation injury management, with implications for both clinical practice and broader scenarios involving mass radiation exposure.

### Supplementary Information

**Additional file 1**. **Supplementary Fig. 1: Visual Comparison of Diverse Treatments in Irradiated Mice Prophylactic Treatment Group.** C57BL/6 mice were irradiated using a 40Gy dose. Changes in the skin architecture and appearance were documented up to day 42 post-irradiation. (A) Saline control group, (B) C57BL/6 ASCs treated mice, (C) metformin-treated mice, (D) ASCs and metformin-treated mice (E) FVB ASCs treated mice, and (F) FVB ASCs and metformin-treated mice. **Supplementary Fig. 2: Histological Comparison of Diverse Treatments in Irradiated Mice.** Masson’s Trichrome stained section from individual mice skin harvest on day 42 post-irradiation. (A) Saline control group, (B) C57BL/6 ASCs treated mice, (C) metformin-treated mice, (D) ASCs and metformin-treated mice (E) FVB ASCs treated mice, and (F) FVB ASCs and metformin-treated mice. **Supplementary Fig. 3: Visual Comparison of Diverse Treatments in Irradiated Mice Late onset treatment group.** C57BL/6 mice were irradiated using a 40Gy dose. Changes in the skin architecture and appearance were documented up to day 42 post-irradiation. (A) Saline control group, (B) C57BL/6 ASCs treated mice, (C) metformin-treated mice, (D) ASCs and metformin-treated mice (E) FVB ASCs treated mice, and (F) FVB ASCs and metformin-treated mice.

## Data Availability

All data reported in this paper are available from the corresponding author upon request.
