# Supplementary material for: Metformin and adipose-derived stem cell combination therapy alleviates radiation-induced skin fibrosis in mice
Source: Stem Cell Res Ther. 2024 Jan 8;15:13. doi: 10.1186/s13287-023-03627-7 (PMC10773046; doi:10.1186/s13287-023-03627-7)
Supplement: Supplementary file 1 — Additional file 1. Supplementary Fig. 1: Visual Comparison of Diverse Treatments in Irradiated Mice Prophylactic Treatment Group. C57BL/6 mice were irradiated using a 40Gy dose. Changes in the skin architecture and appearance were documented up to day 42 post-irradiation. (A) Saline control group, (B) C57BL/6 ASCs treated mice, (C) metformin-treated mice, (D) ASCs and metformin-treated mice (E) FVB ASCs treated mice, and (F) FVB ASCs and metformin-treated mice. Supplementary Fig. 2: Histological Comparison of Diverse Treatments in Irradiated Mice. Masson’s Trichrome stained section from individual mice skin harvest on day 42 post-irradiation. (A) Saline control group, (B) C57BL/6 ASCs treated mice, (C) metformin-treated mice, (D) ASCs and metformin-treated mice (E) FVB ASCs treated mice, and (F) FVB ASCs and metformin-treated mice. Supplementary Fig. 3: Visual Comparison of Diverse Treatments in Irradiated Mice Late onset treatment group. C57BL/6 mice were irradiated using a 40Gy dose. Changes in the skin architecture and appearance were documented up to day 42 post-irradiation. (A) Saline control group, (B) C57BL/6 ASCs treated mice, (C) metformin-treated mice, (D) ASCs and metformin-treated mice (E) FVB ASCs treated mice, and (F) FVB ASCs and metformin-treated mice. [file 13287_2023_3627_MOESM1_ESM.pdf]

Irradiated Control

A

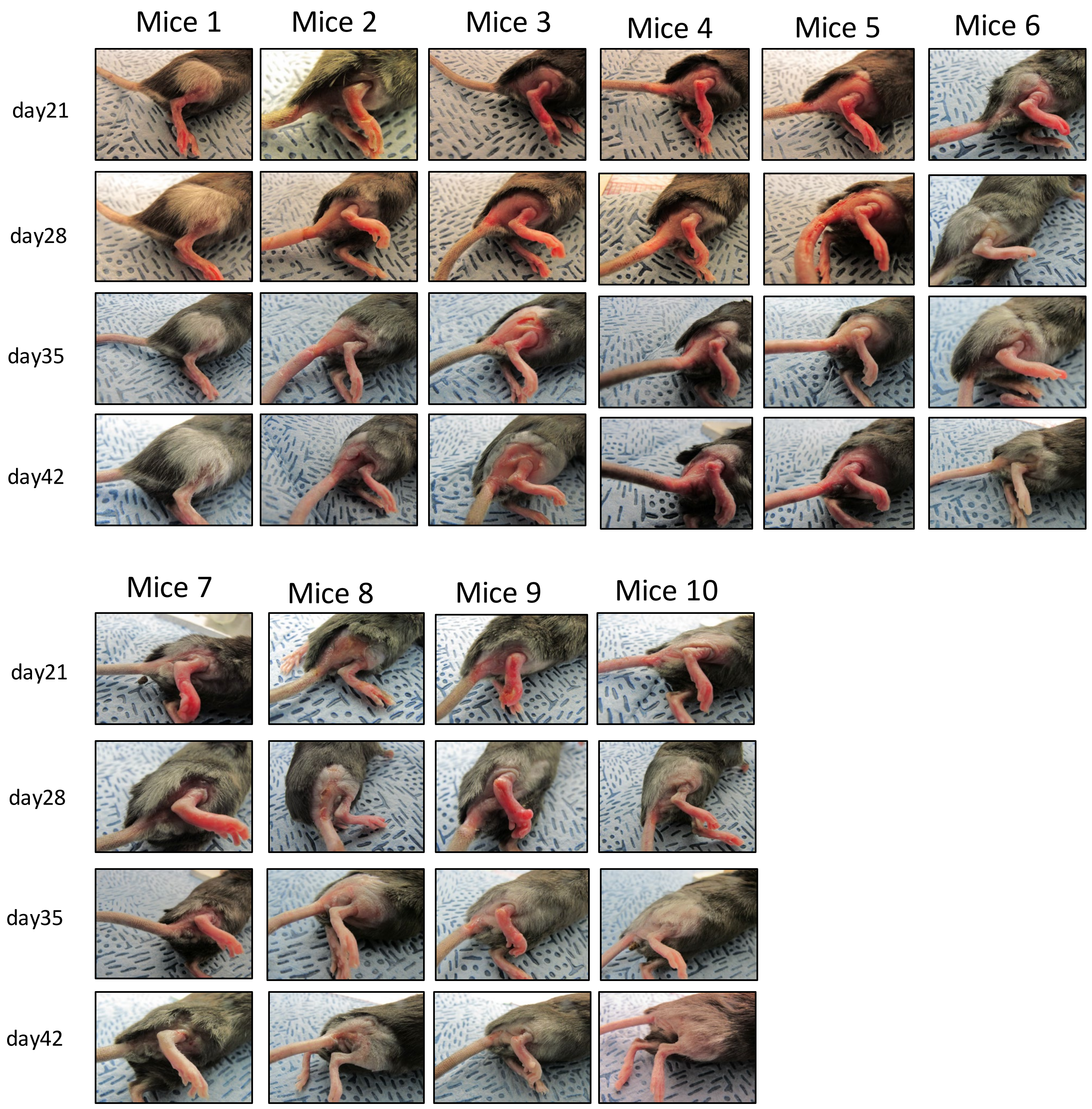

Supplementary Figure 1

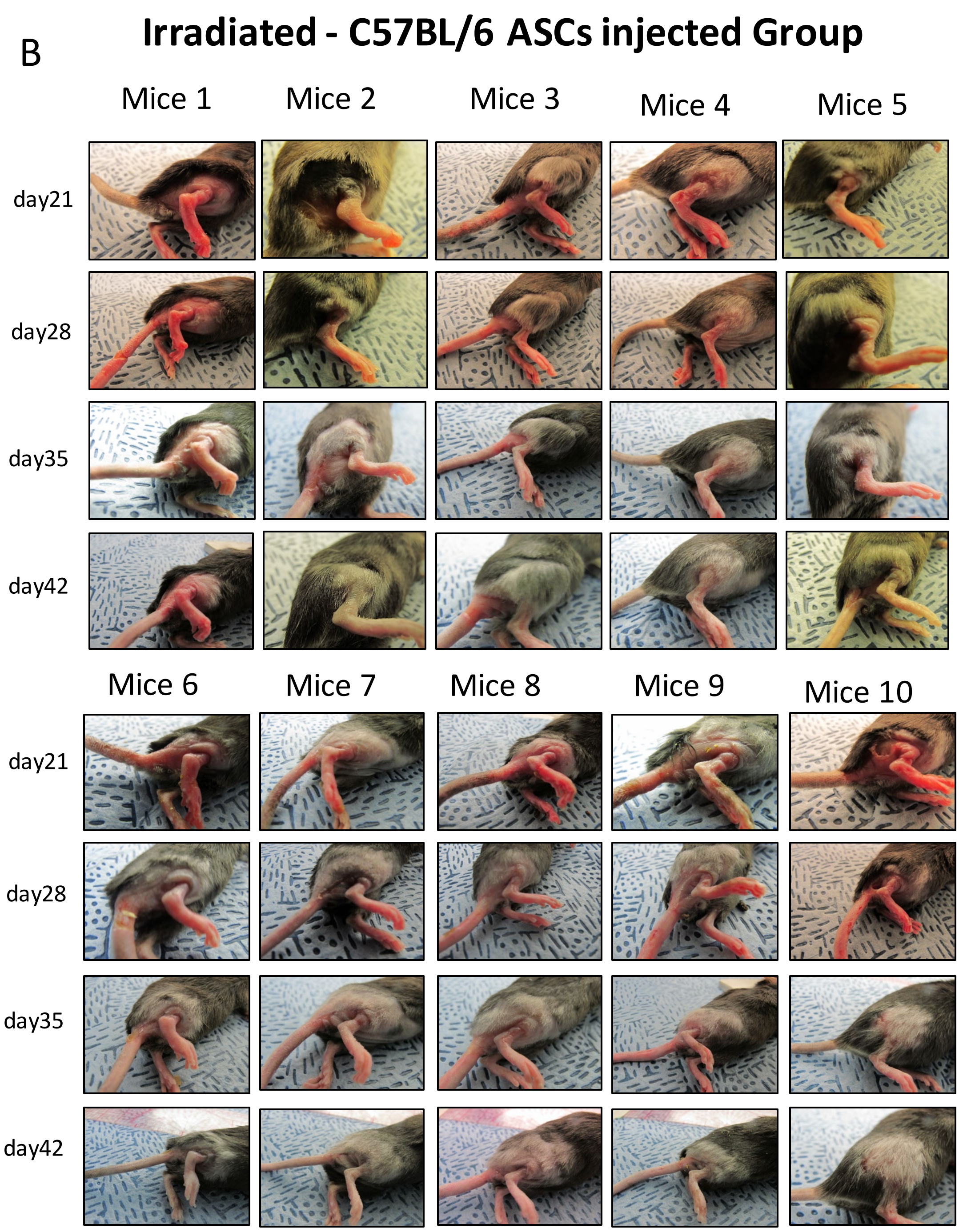

Supplementary Figure 1

Irradiated - Metformin injected Group

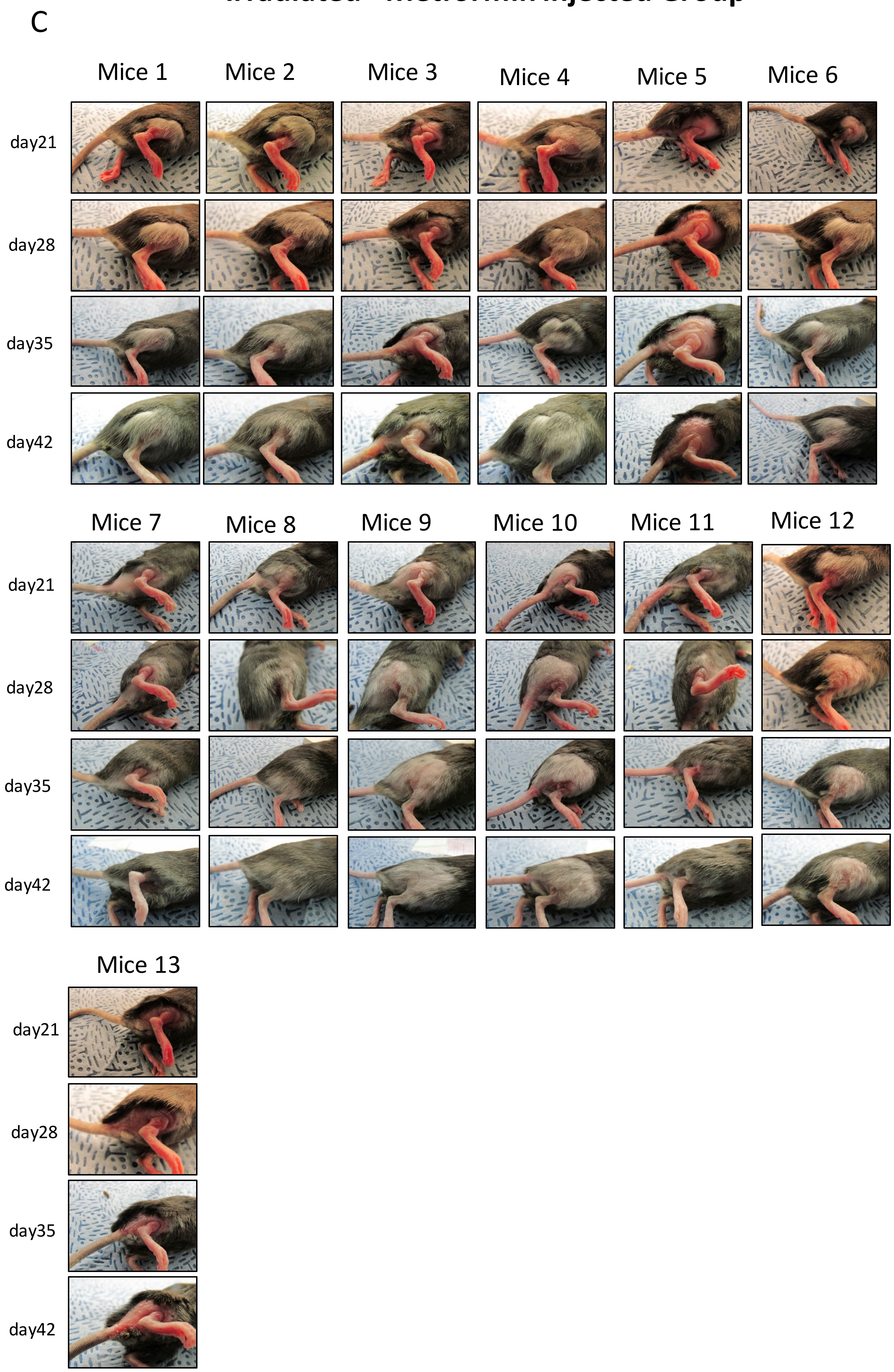

Supplementary Figure 1

Irradiated - C57 BL/6 ASCs + Metformin injected Group

D

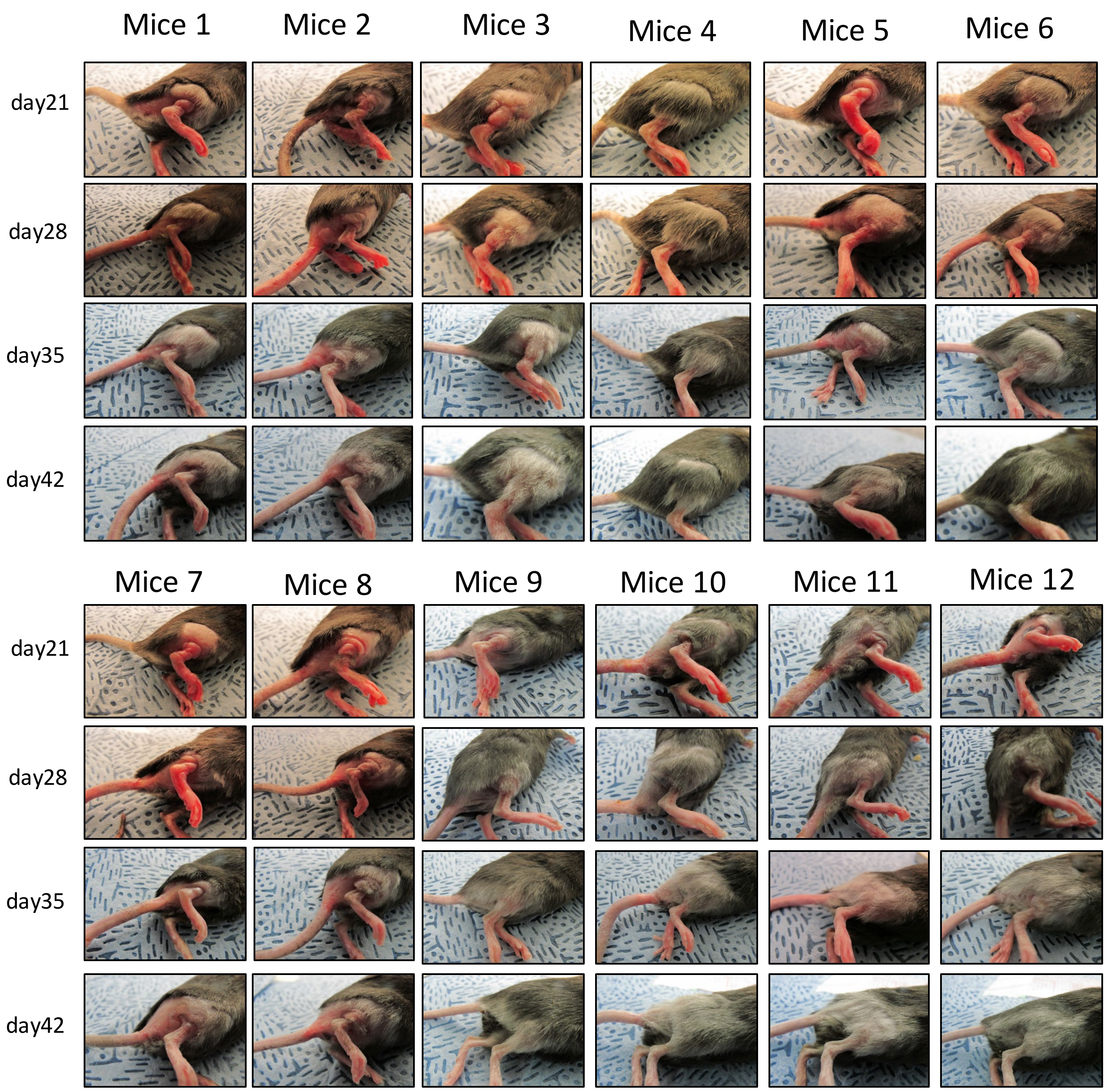

Supplementary Figure 1

Irradiated- FVB ASCs injected Group

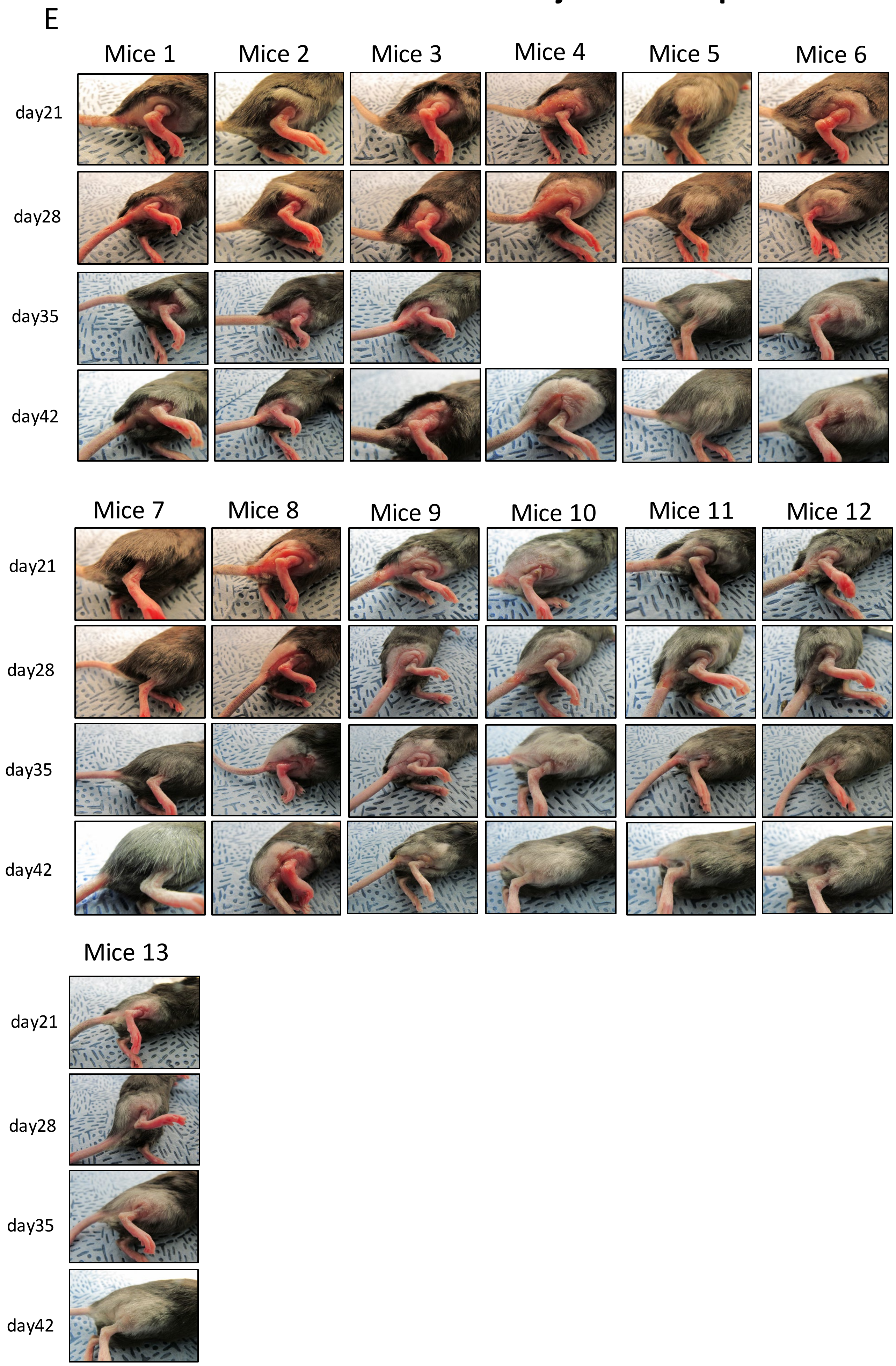

Supplementary Figure 1

F

Irradiated- FVB ASCs + Metformin injected Group

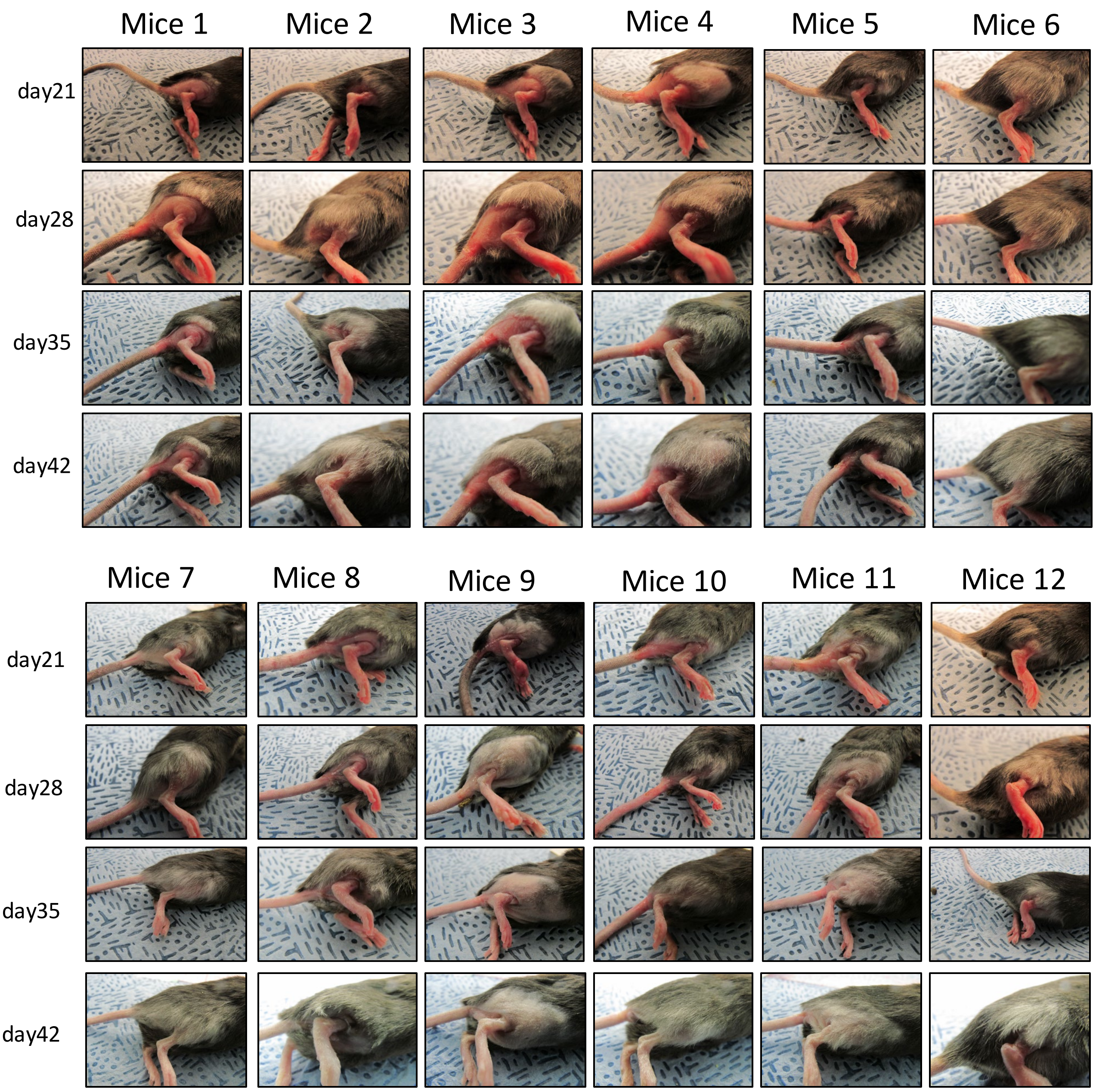

Supplementary Figure 1

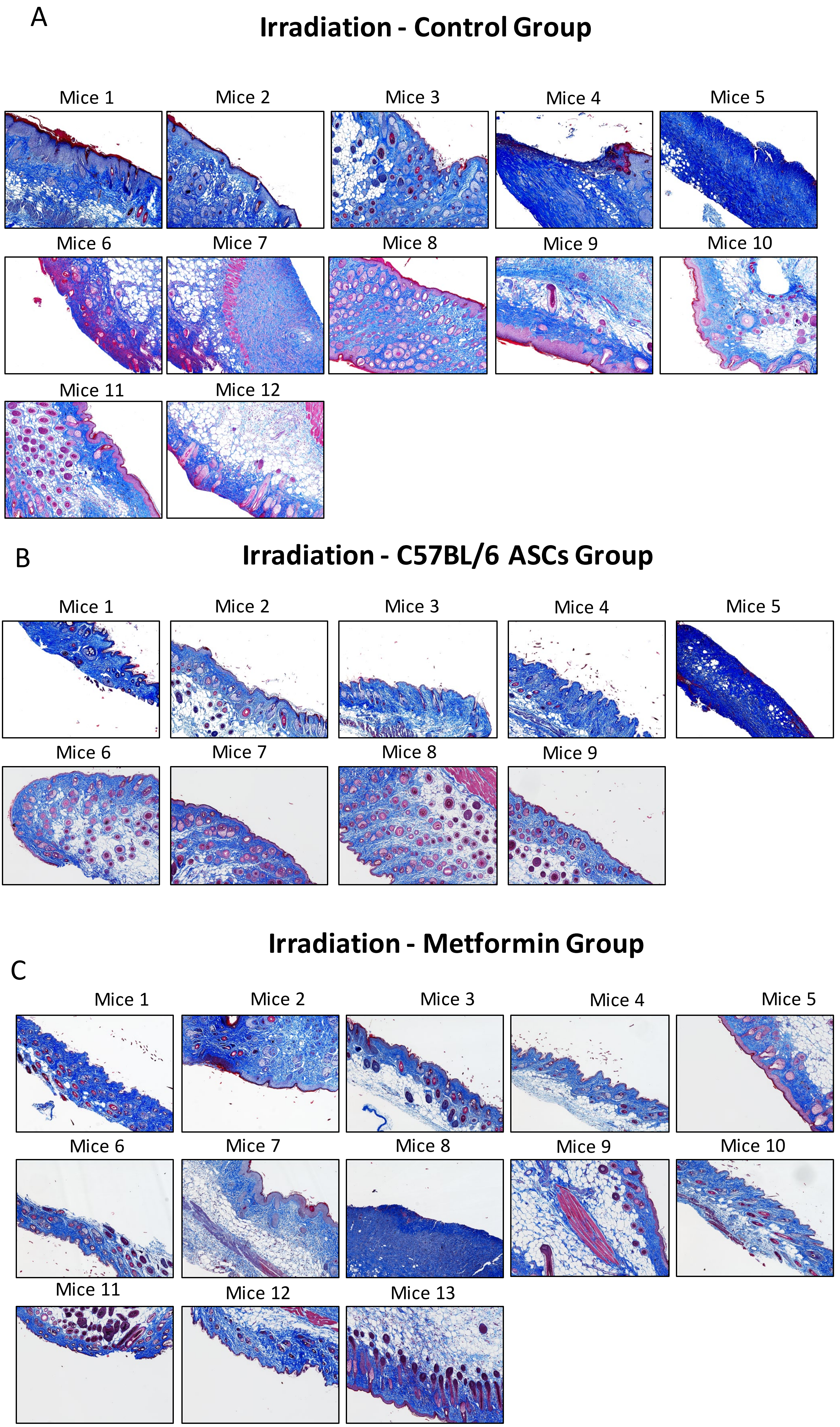

Supplementary Figure 2

D

Irradiation - C57BL/6+Metformin Group

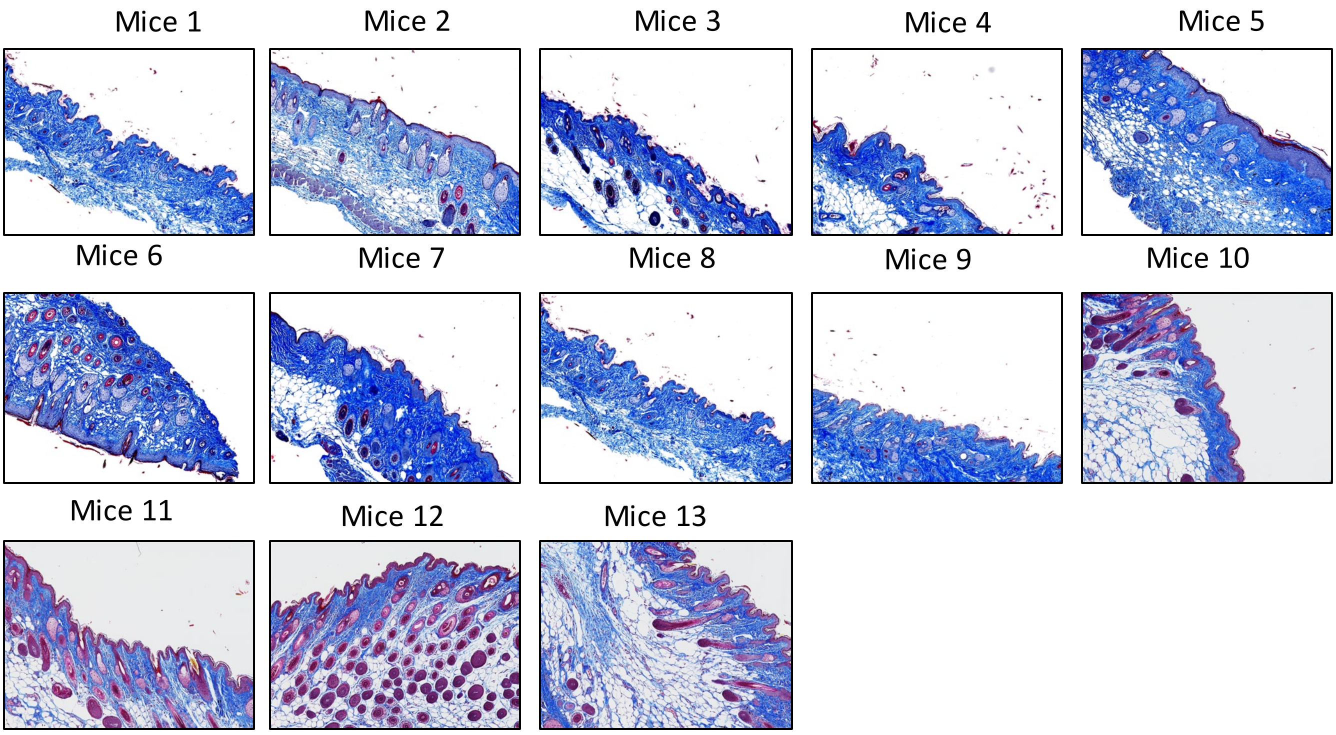

E

Irradiation - FVB ASCs Group

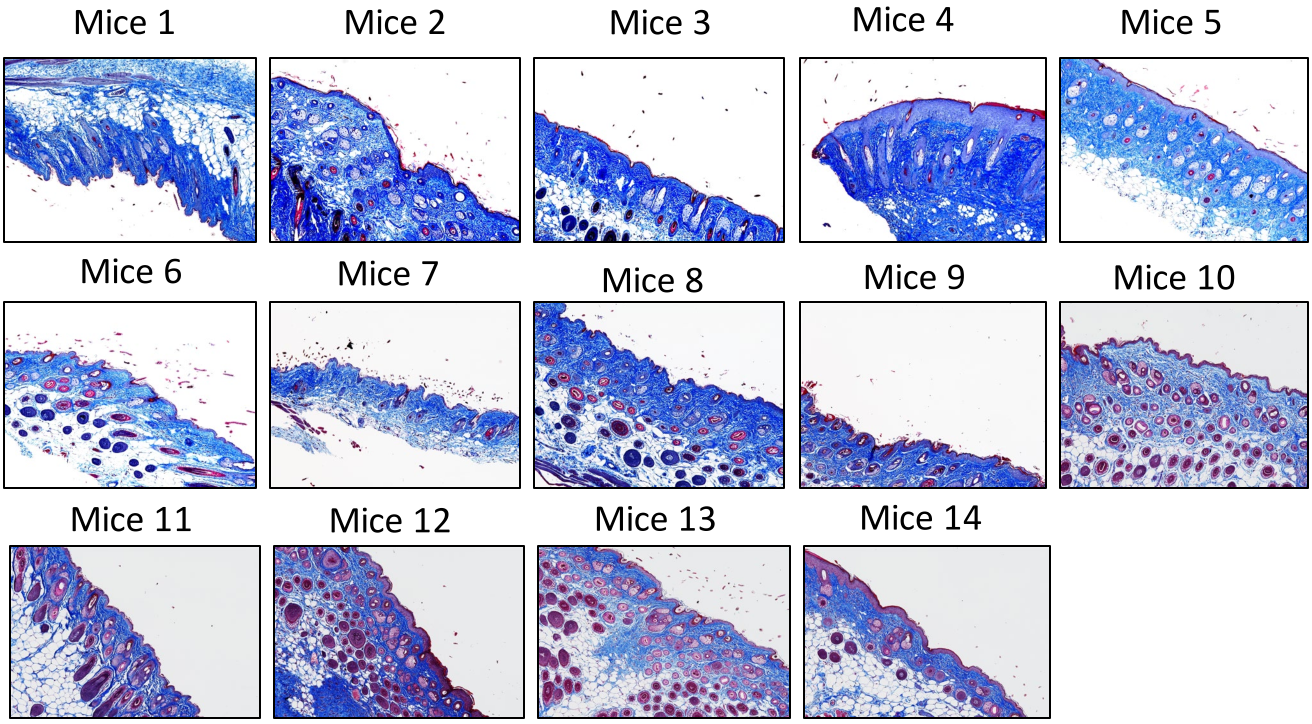

F

Irradiation- FVB ASCs+ Metformin Group

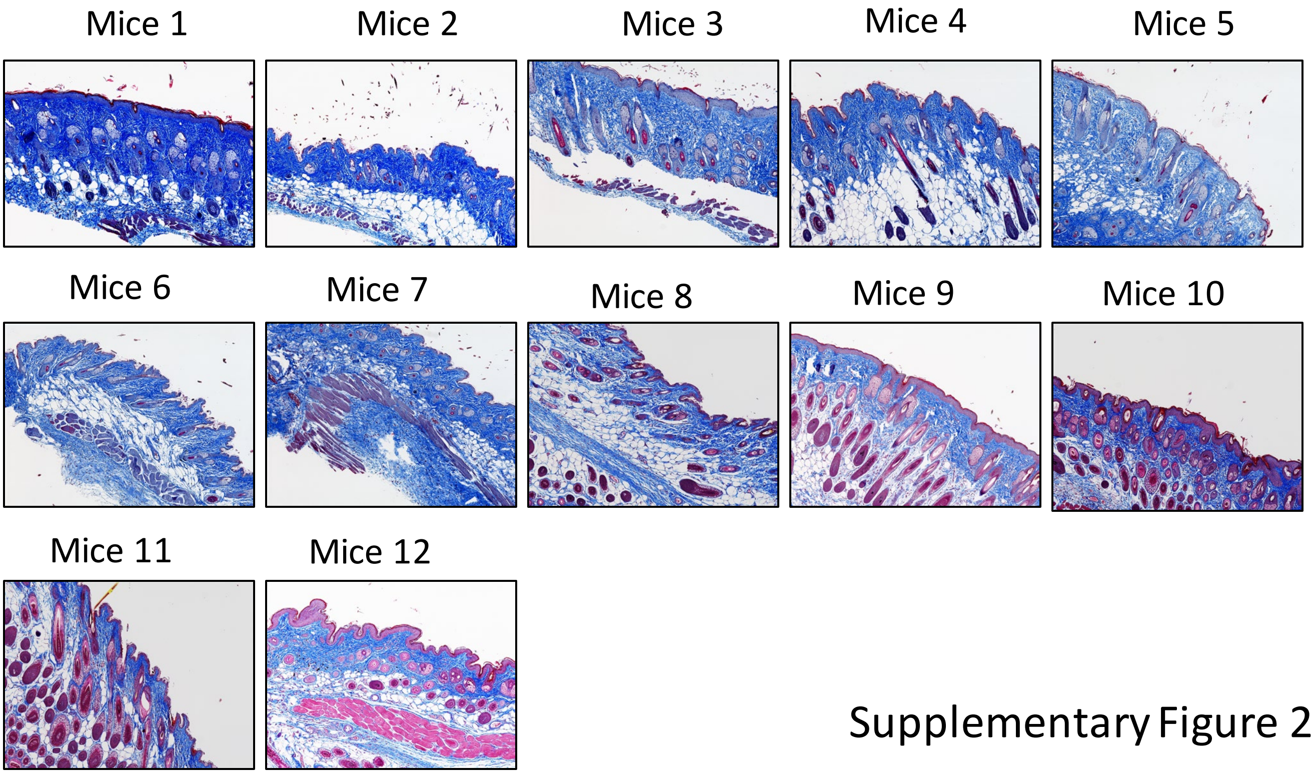

Supplementary Figure 2

A

Irradiated Control

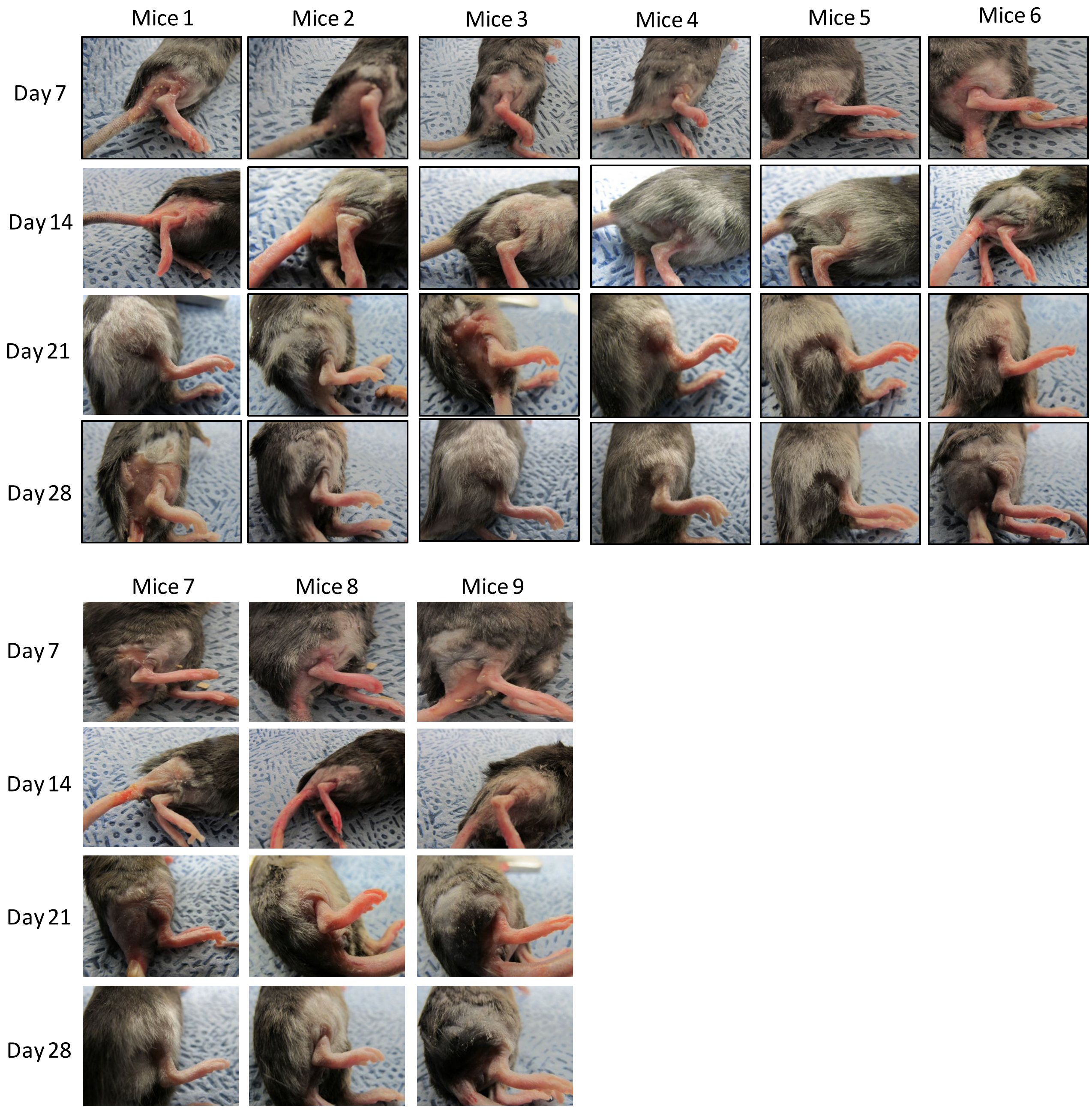

Supplementary Figure 3

Irradiated - C57BL/6 ASCs injected Group

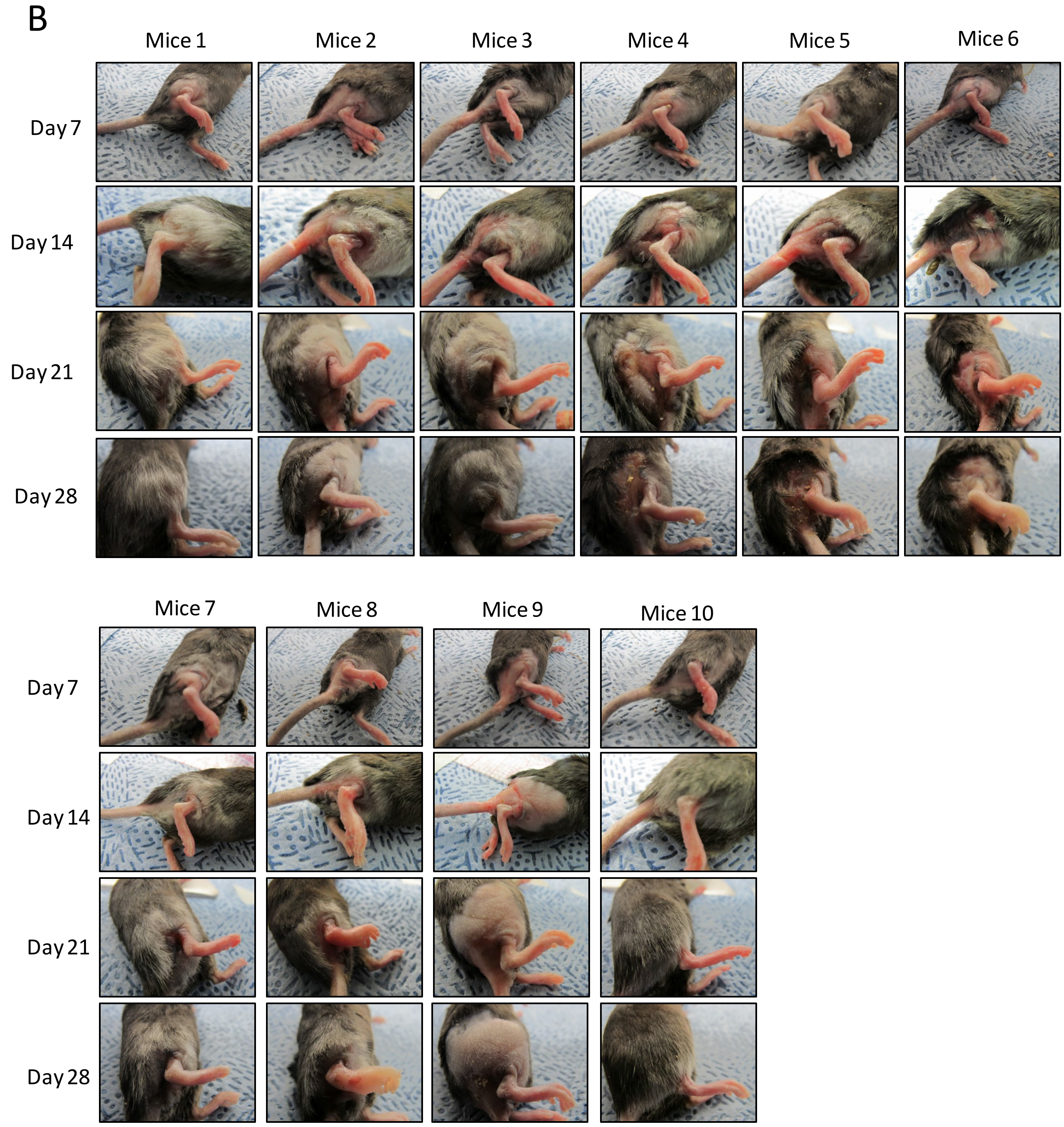

Supplementary Figure 3

Irradiated - Metformin injected Group

C

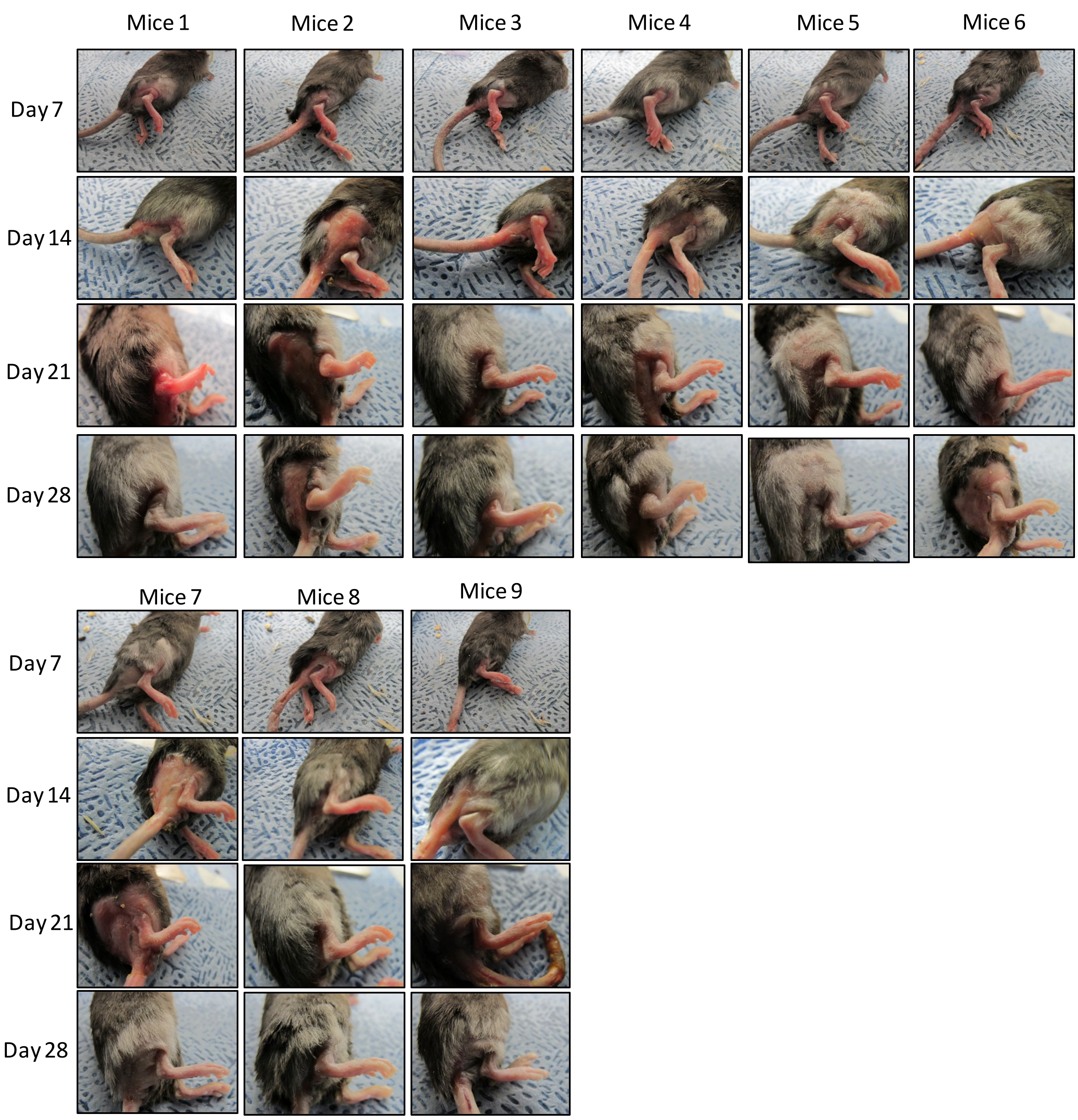

Supplementary Figure 3

Irradiated - C57BL/6 ASCs + Metformin injected Group

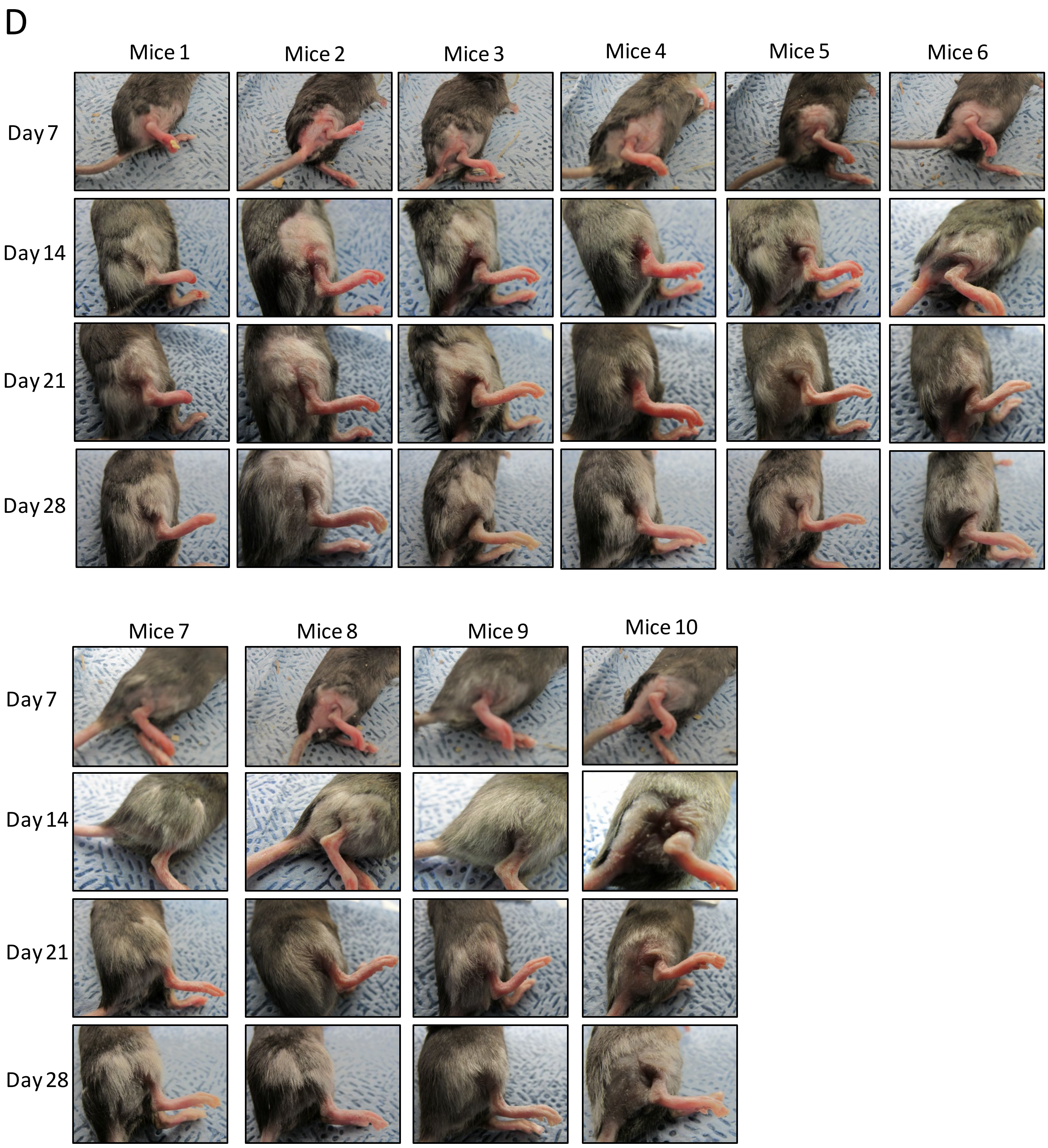

Supplementary Figure 3

Irradiated - FVB ASCs injected Group

E

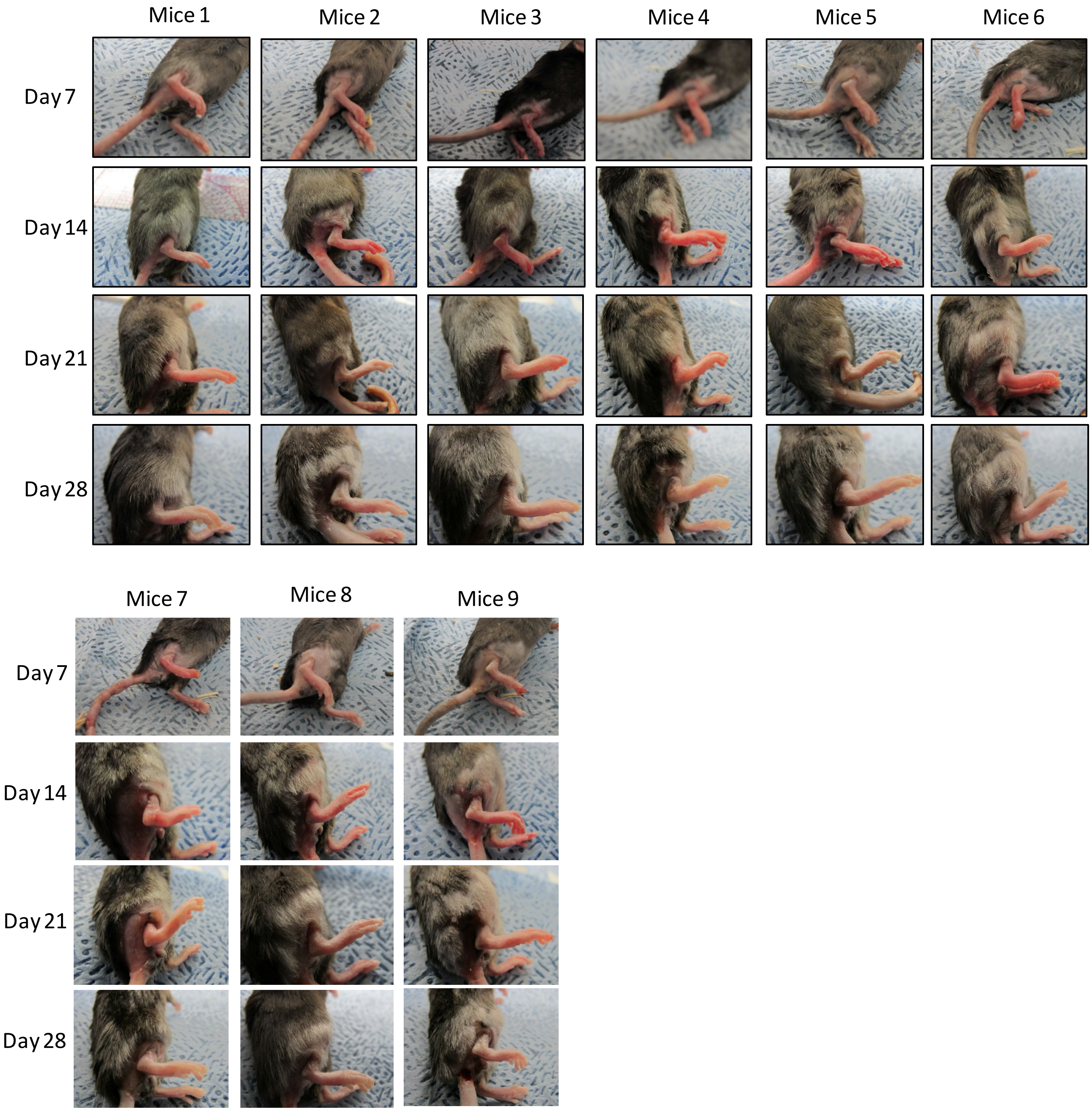

Supplementary Figure 3

Irradiated - FVB ASCs + Metformin injected Group

F

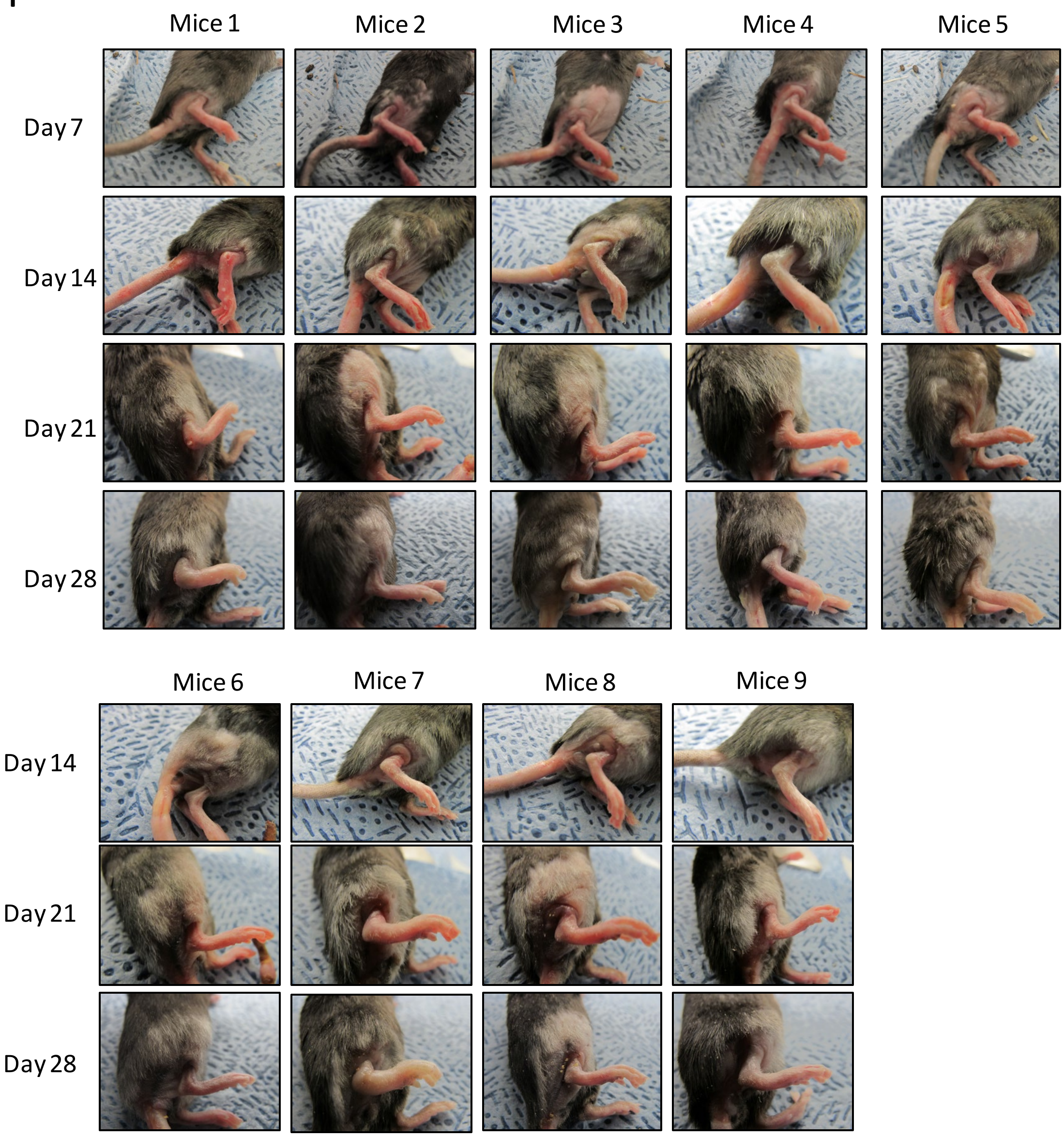

Supplementary Figure 3
